# Supplementary material for: An mTORC1-mediated negative feedback loop constrains amino acid-induced FLCN-Rag activation in renal cells with TSC2 loss
Source: Nat Commun. 2022 Nov 10;13:6808. doi: 10.1038/s41467-022-34617-7 (PMC9649702; doi:10.1038/s41467-022-34617-7)
Supplement: Supplementary file 1 — Supplementary Information [file 41467_2022_34617_MOESM1_ESM.pdf]

Supplementary Figure 1

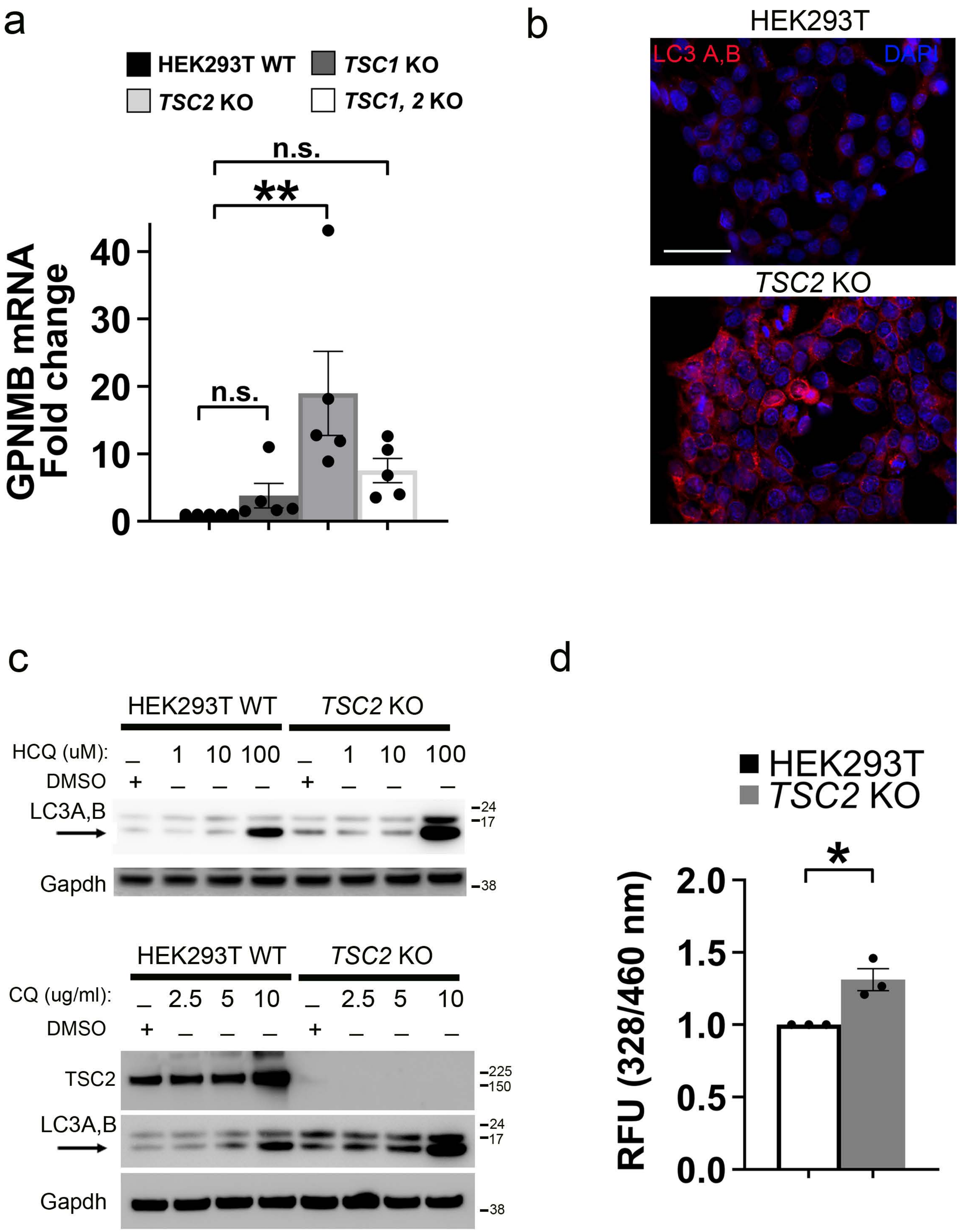

Supplementary Figure Legends

Supplementary Figure S1: *mTORC1 hyperactivation via TSC2 loss drives lysosomal biogenesis in murine and human renal tumor cells*. (a) Quantitative real time PCR (qRT-PCR) for GPNMB transcripts in *TSC1, TSC2* and *TSC1/2* KO cells compared to WT controls. n= 5 independent biological replicates. Graphs are presented as mean values $\pm$ SEM. Statistical analyses were performed using one-way ANOVA with Dunnett's test for multiple comparisons. p=0.0045 (WT vs *TSC2* KO) (also see Figure 1c). **(B)** Indirect immunofluorescence of HEK293T WT and *TSC2* KO cells for detection of LC3 A, B puncta using an anti-LC3 A, B antibody (Scale bar=100 µm). **(C)** Immunoblotting of whole cell lysates from WT and *TSC2* KO cells treated with increasing doses of Hydroxychloroquine (HCQ, top panel) or Chloroquine (CQ, bottom panel) for 24 hrs prior to lysis. **(D)** Lysosomal activity, as measured by fluorometric analyses of cathepsin D activity using a Cathepsin D activity assay kit, is significantly increased in *TSC2* KO cells compared to WT controls. n= 3 independent biological replicates. Graphs are presented as mean values $\pm$ SEM. Statistical analyses were performed using two-tailed Students *t*-test. p=0.0146. Source data are provided as a Source Data file.

Supplementary Figure 2

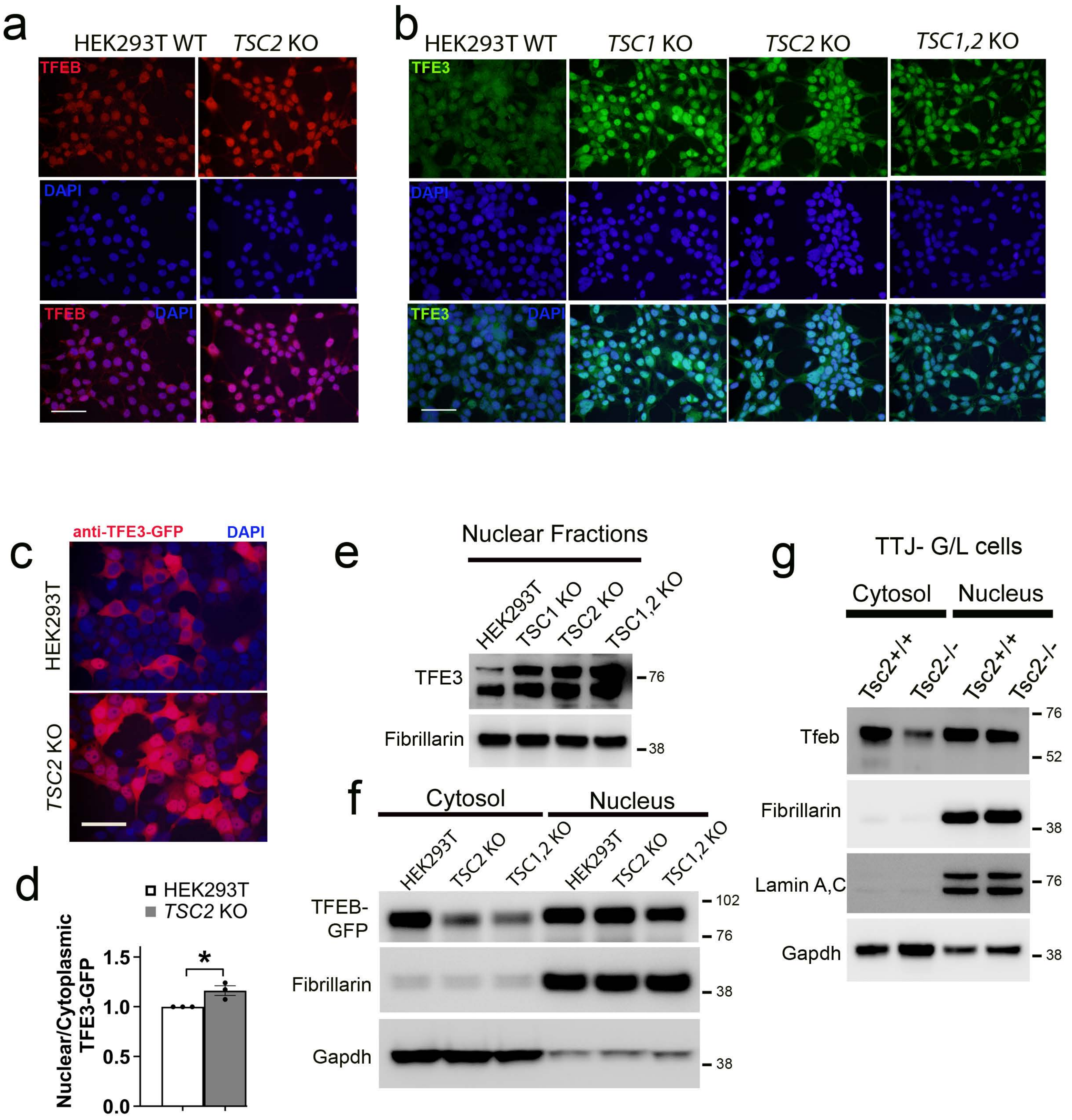

Supplementary Figure S2: ***MiT/TFE nuclear localization and transcriptional activity is increased in vitro with TSC2 loss*****. (a)** Representative images of HEK293T WT and *TSC2* KO cells immune-stained for endogenous TFEB (also see figure 2a) (Scale bar=100 µm). **(b)** Representative images of HEK293T WT, *TSC1* KO, *TSC2* KO and *TSC1/2* KO cells immune-stained for endogenous TFE3 (also see figure 2b) (Scale bar=100 µm). **(c)** Representative images of WT and *TSC2* KO cells transiently transfected with a TFE3-GFP plasmid for 24 hrs, and with indirect immunofluorescence using an anti-GFP antibody (Scale bar=100 µm). **(d)** Quantification of nuclear/cytoplasmic TFE3-GFP fluorescence from experiments in (c) n=3 independent biological replicates, counting 645 cells per replicate. Graphs are presented as mean values ± SEM. Statistical analyses were performed using two-tailed Students *t*-test. p=0.0281. **(e)** TFE3 expression in nuclear-fraction immunoblots of *TSC1* KO, *TSC2* KO and *TSC1/2* KO cells compared to WT controls. **(f)** Immunoblotting of cytosolic and nuclear fractions from HEK293T WT, *TSC2* KO and *TSC1/2* KO cells transiently transfected with a TFEB-GFP plasmid for 24 hrs. **(g)** TFEB expression in nuclear-fraction immunoblots of *Tsc2*-null, TTJ-parental cells compared to their *Tsc2* re-expressing, wild-type counterparts. Source data are provided as a Source Data file.

Supplementary Figure 3

a

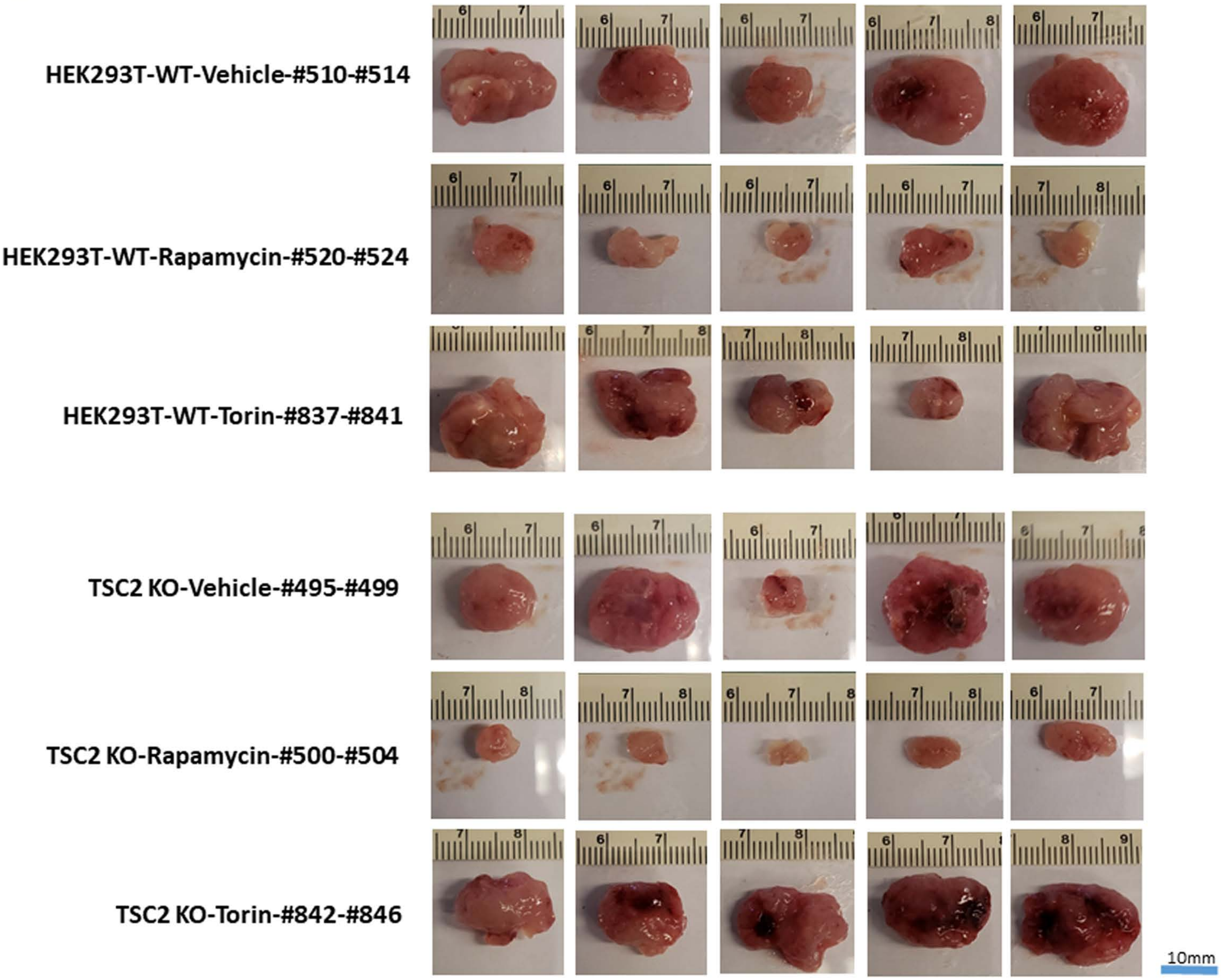

b

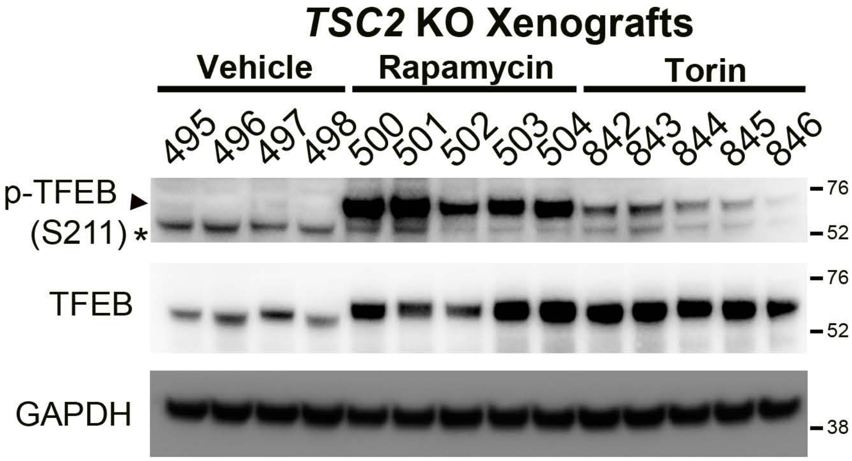

Supplementary Figure S3: ***MiT/TFE nuclear localization and transcriptional activity is increased in vitro with TSC2 loss*****.** **(a)** Representative gross images of tumor xenografts from WT and *TSC2* KO xenografts in NSG mice treated with vehicle, rapamycin or torin (from experiments in Figure 2 k, l). **(b)** Immunoblotting of tumor lysates from *TSC2* KO xenografts (from experiments in Figure 2 k, l) treated with vehicle, rapamycin or torin. Source data are provided as a Source Data file.

Supplementary Figure 4

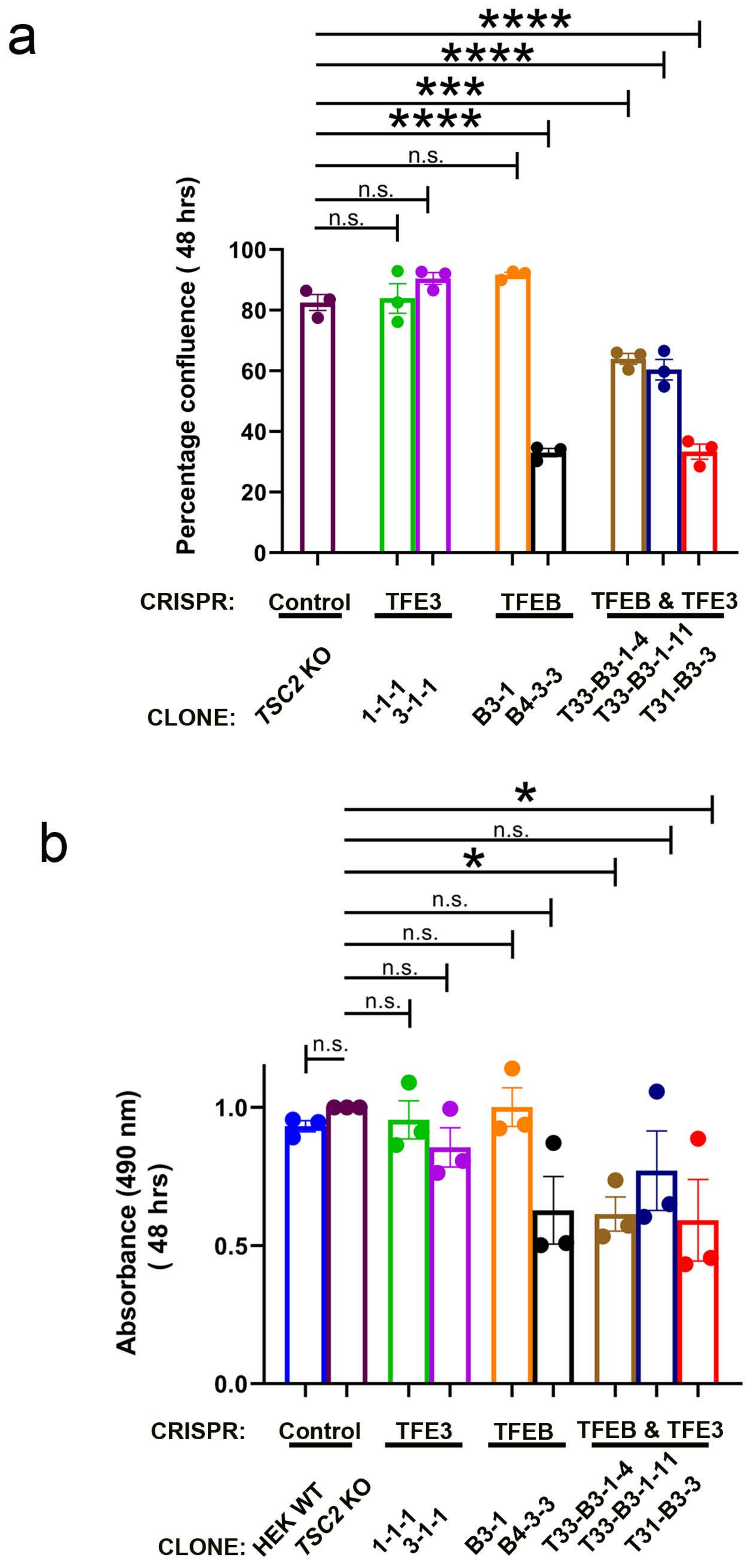

Supplementary Figure S4: *Genomic inactivation of* TFEB *and* TFE3 *decreases proliferation of TSC2 KO cells and xenografts.* (a) *In vitro* growth of *TSC2* KO cells with single or combined genomic inactivation of *TFEB* and *TFE3* at 48 hrs of growth assessed by IncuCyte ZOOM live-cell imaging. n= 3 independent biological replicates. Graphs are presented as mean values ± SEM. Statistical analyses were performed using one-way ANOVA with Dunnett's test for multiple comparisons. ***$p < 0.001$, ****$p < 0.0001$. **(b)** *In vitro* growth of WT and *TSC2* KO cells with single or combined genomic inactivation of *TFEB* and *TFE3* at 48 hrs of growth assessed by colorimetric analyses of viable cells using the CellTiter 96® Aqueous One Solution Cell Proliferation Assay (MTS). n= 3 independent biological replicates. Graphs are presented as mean values ± SEM. Statistical analyses were performed using one-way ANOVA with Dunnett's test for multiple comparisons. $p = 0.0475$ (*TSC2* KO vs T33-B3-1-4) and $p = 0.0336$ (*TSC2* KO vs T31-B3-3). Source data are provided as a Source Data file.

Supplementary Figure 5

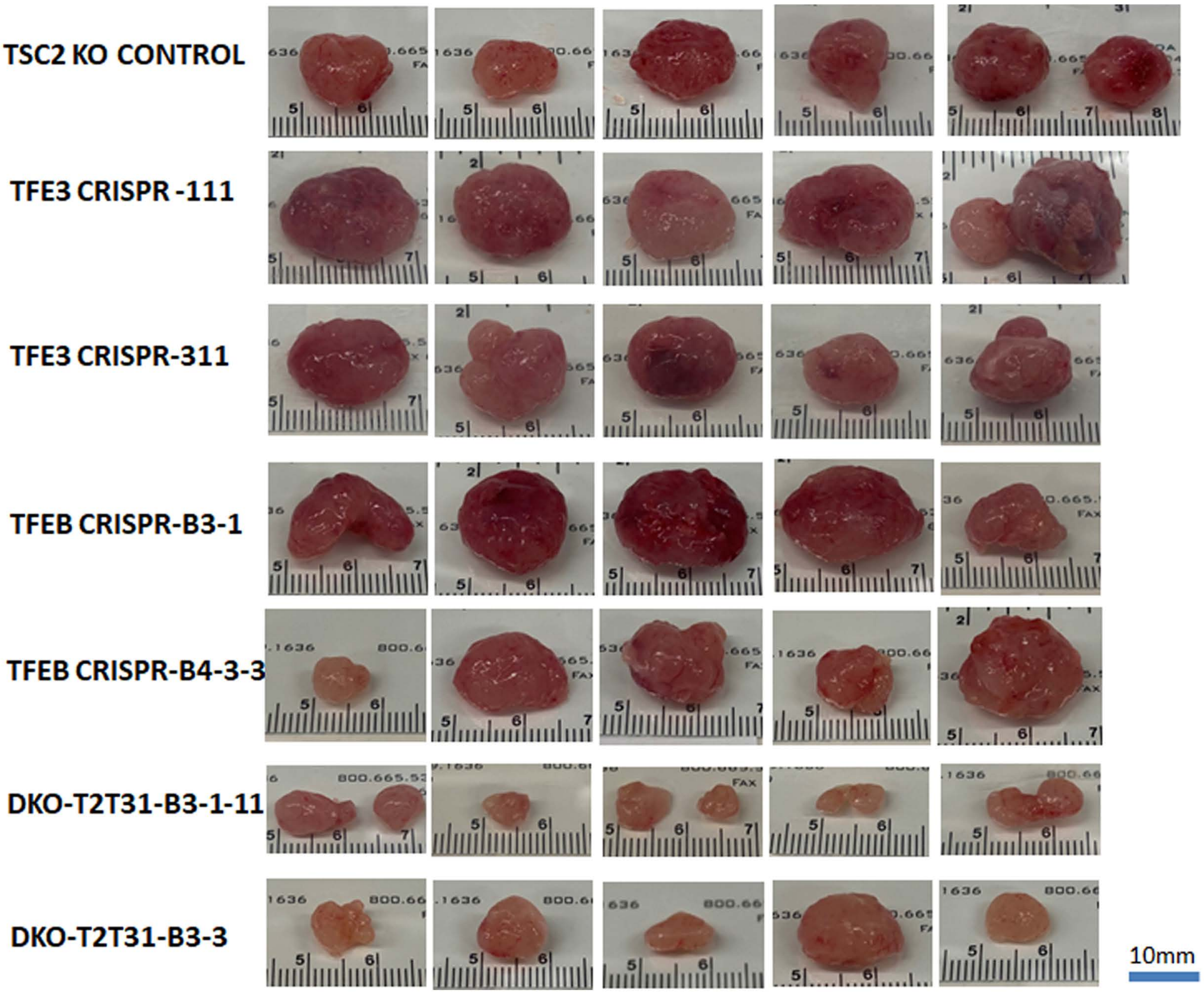

Supplementary Figure S5: ***Genomic inactivation of TFEB and TFE3 decreases proliferation of TSC2 KO cells and xenografts.*** Representative gross images of tumor xenografts from *TSC2* KO parental control CRISPR cells and cells with genomic deletion of *TFEB, TFE3* or *TFEB and TFE3.*

Supplementary Figure 6

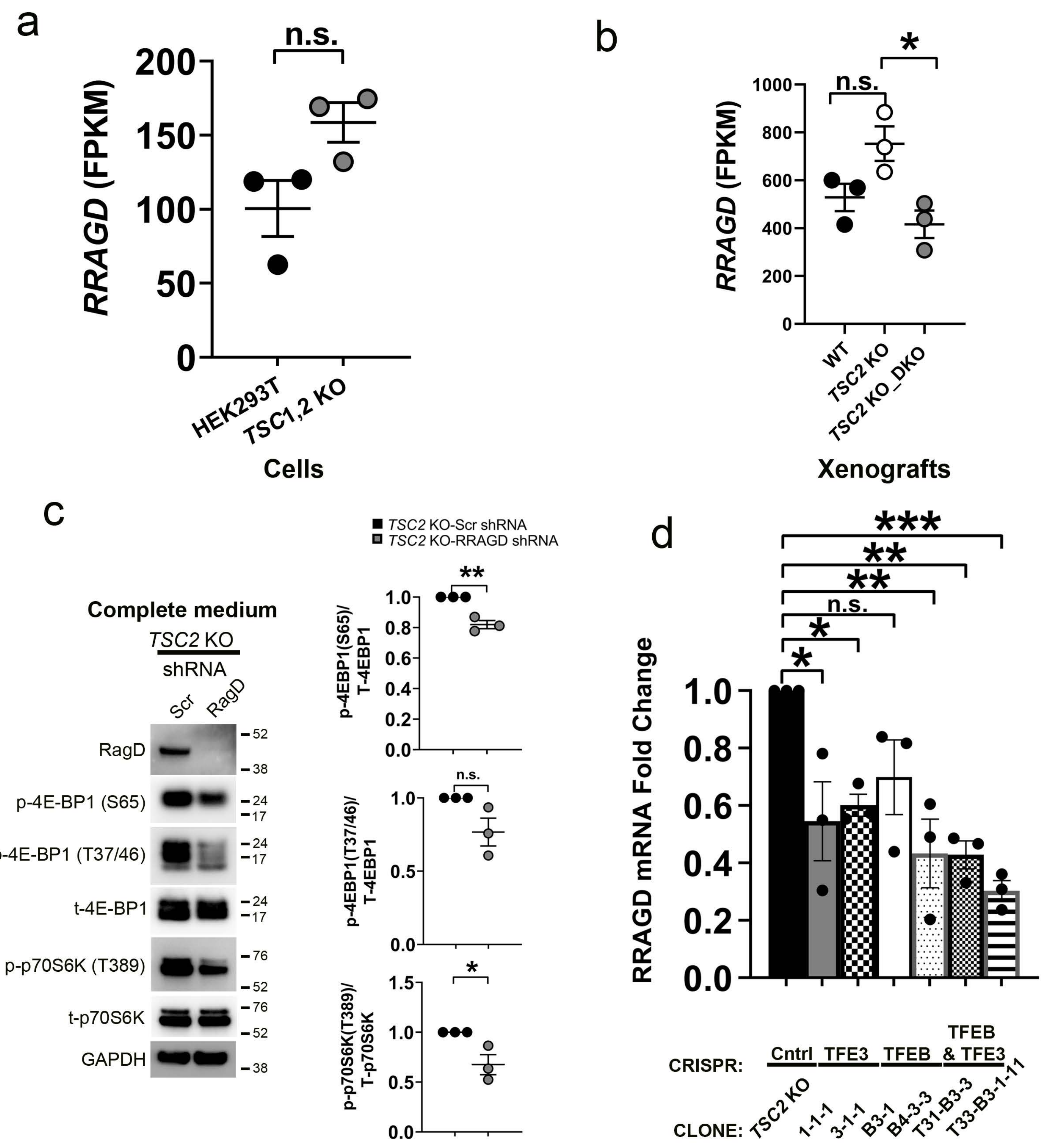

Supplementary Figure S6: ***Genomic inactivation of TFEB and TFE3 decreases proliferation of TSC2 KO cells and xenografts.*** **(a)** Expression of *RRAGD (*FPKM) in HEK293T WT cells vs *TSC1/2* KO cells. n= 3 independent biological replicates*.* Scatter plots are presented as mean values + SEM. Statistical analyses were performed using two-tailed Students *t*-test. **(b)** Expression of *RRAGD (*FPKM) in xenografts from WT control CRISPR cells, *TSC2* KO control CRISPR cells and *TSC2* KO cells with genomic deletion of *TFEB* and *TFE3.* n= 3 independent biological replicates*.* Scatter plots are presented as mean values + SEM. Statistical analyses were performed using one-way ANOVA with Dunnett's test for multiple comparisons. *p*= 0.016 (*TSC2* KO vs *TSC2* KO_DKO). **(c)** Immunoblotting of *TSC2* KO scrambled control shRNA and *RRAGD* shRNA-transfected cells for mTORC1 activation markers ((p-4EBP1(S65), p-4EBP1 (T37/46) and p-p70S6K (T389)) (left panels). Densitometry quantification of normalized p-4EBP1 (S65), p-4EBP1 (T37/46) and p-p70S6K (T389) band intensities (right panels). n= 3 independent biological replicates*.* Scatter plots are presented as mean values + SEM. Statistical analyses were performed using two-tailed Students *t*-test. *p*= 0.0025 (p-4EBP1(S65)) and *p*= 0.0318 (p-p70S6K (T389)). **(d)** qRT-PCR for relative *RRAGD* gene expression in *TSC2* KO HEK293T cells with or without genomic deletion of *TFE3, TFEB* or *TFEB and TFE3.* n= 3 independent biological replicates*.* Scatter plots are presented as mean values + SEM. Statistical analyses were performed using one-way ANOVA with Dunnett's test for multiple comparisons. **p*< 0.05, ***p*< 0.01, ****p*< 0.001. Source data are provided as a Source Data file.

Supplementary Figure 7

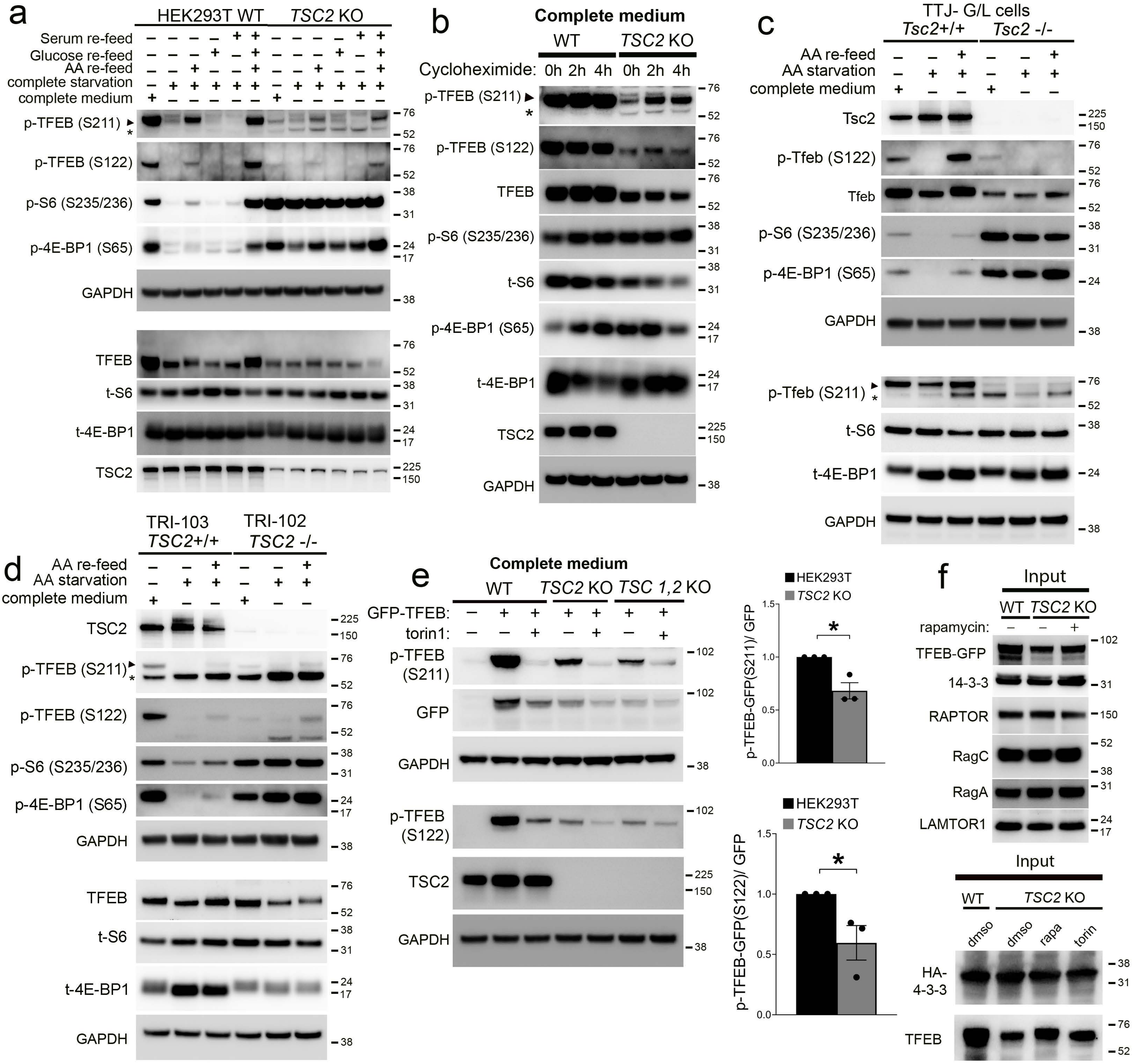

Supplementary Figure S7: *TSC2 loss decreases phosphorylation of TFEB at canonical mTORC1 sites in an amino-acid dependent and rapamycin-sensitive manner.* **(a)** Immunoblotting of whole cell lysates from HEK293T WT and *TSC2* KO cells maintained in complete growth media and left untreated, nutrient starved (90 min) or starved and re-stimulated (30 min) with either amino acids, glucose, dialyzed serum or a combination of the three. TFEB, t-S6, t-4EBP1 and TSC2 are non-contemporaneous immunoblots from the same biological replicate. **(b)** Immunoblotting of whole cell lysates from WT and *TSC2* KO cells treated with Cycloheximide (CHX) (50 µg/ml) for 2 or 4 hrs prior to lysis. **(c)** Immunoblotting of whole cell lysates from *Tsc2*-null, TTJ-parental cells and their *Tsc2* re-expressing, wild-type counterparts, maintained in complete growth media, and left untreated, amino acids starved (90 min) or starved and re-stimulated with amino acids (30 min). p-Tfeb (S211), t-S6, t-4EBP1 and GAPDH are non-contemporaneous immunoblots from the same biological replicate. **(d)** Immunoblotting of whole cell lysates from *TSC2*-null, TRI-102 cells and their *TSC2* re-expressing, wild-type counterparts (TRI-103 cells) maintained in complete growth media and left untreated, amino acids starved (90 min) or starved and re-stimulated with amino acids (30 min), followed by immunoblotting of lysates with the indicated antibodies. TFEB, t-S6, t-4EBP1 and GAPDH are non-contemporaneous immunoblots from the same biological replicate. **(e)** Immunoblotting of whole cell lysates from HEK293T WT, *TSC2* KO and *TSC1/2* KO cells transiently transfected with a TFEB-GFP plasmid for 24 hrs, and treated with vehicle control or torin1 (1 µM) for 2 hrs. The plots on the right show densitometry quantification and normalized p-TFEB/TFEB ratios of TFEB-GFP transfected, HEK293T WT and *TSC2* KO cells. n= 3 independent biological replicates. Graphs are presented as mean values

± SEM. Statistical analyses were performed using two-tailed Students *t*-test. $p= 0.0141$ (p-TFEB GFP (S211)) and $p= 0.0476$ (p-TFEB GFP (S122)). p-TFEB (S122), TSC2 and GAPDH are non-contemporaneous immunoblots from the same biological replicate. **(f)** Immunoblotting of whole cell lysates from immunoprecipitation experiments in Figure 5e. Source data are provided as a Source Data file.

Supplementary Figure 8

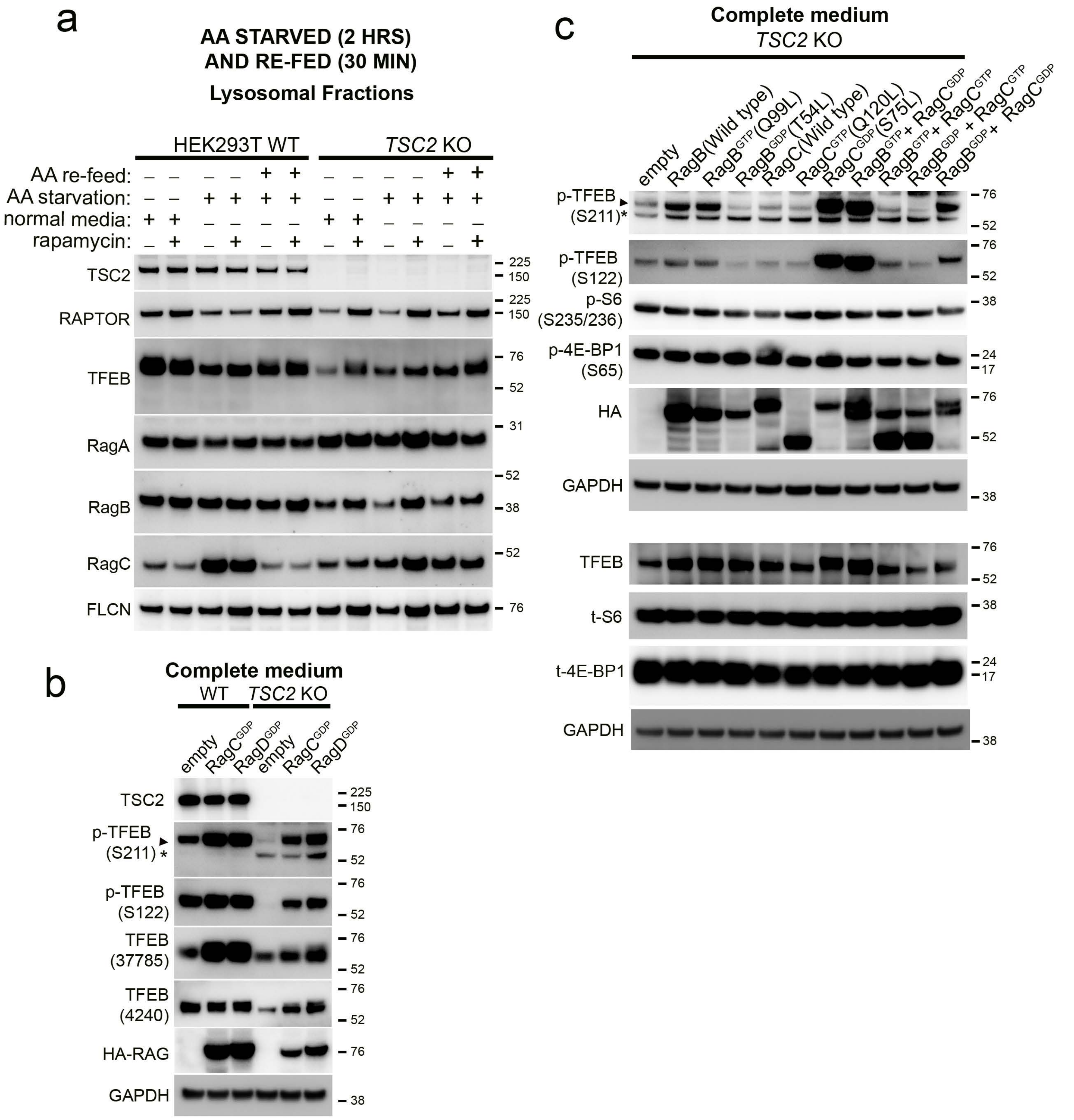

Supplementary Figure S8: ***Active RagC/D heterodimers rescue TFEB lysosomal localization, phosphorylation and cytoplasmic retention in cells with TSC2 loss.*** **(a)** HEK293T WT and *TSC2* KO cells in complete growth media, were either left untreated (lanes 1,2,7,8), amino acids starved for 90 min (lanes 3,4,9,10), or starved and re-stimulated with amino acids for 30 min (lanes 5,6,11,12). Cells were either treated with vehicle control (lanes 1, 3, 5, 7, 9, 11) or rapamycin (200nM; 2hrs) (lanes 2, 4, 6, 8, 10, 12), prior to lysosomal fractionation and immuno-blotting of lysosomal fractions with the indicated antibodies. Rag A expression was unchanged and used as a loading control. **(b)** WT and *TSC2* KO cells were transiently transfected with control vector or HA-tagged, active $RagC^{GDP}$ (S75L) or active $RagD^{GDP}$ (S77L) for 24 hrs prior to lysis and immunoblotting (also see Figure 6b). **(c)** Immunoblotting of *TSC2* KO cells transiently transfected with control vector or HA-tagged, WT, active or inactive Rag mutants as indicated, for 24 hrs. TFEB, t-S6, t-4EBP1 and GAPDH are non-contemporaneous immunoblots from the same biological replicate. Source data are provided as a Source Data file.

Supplementary Figure 9

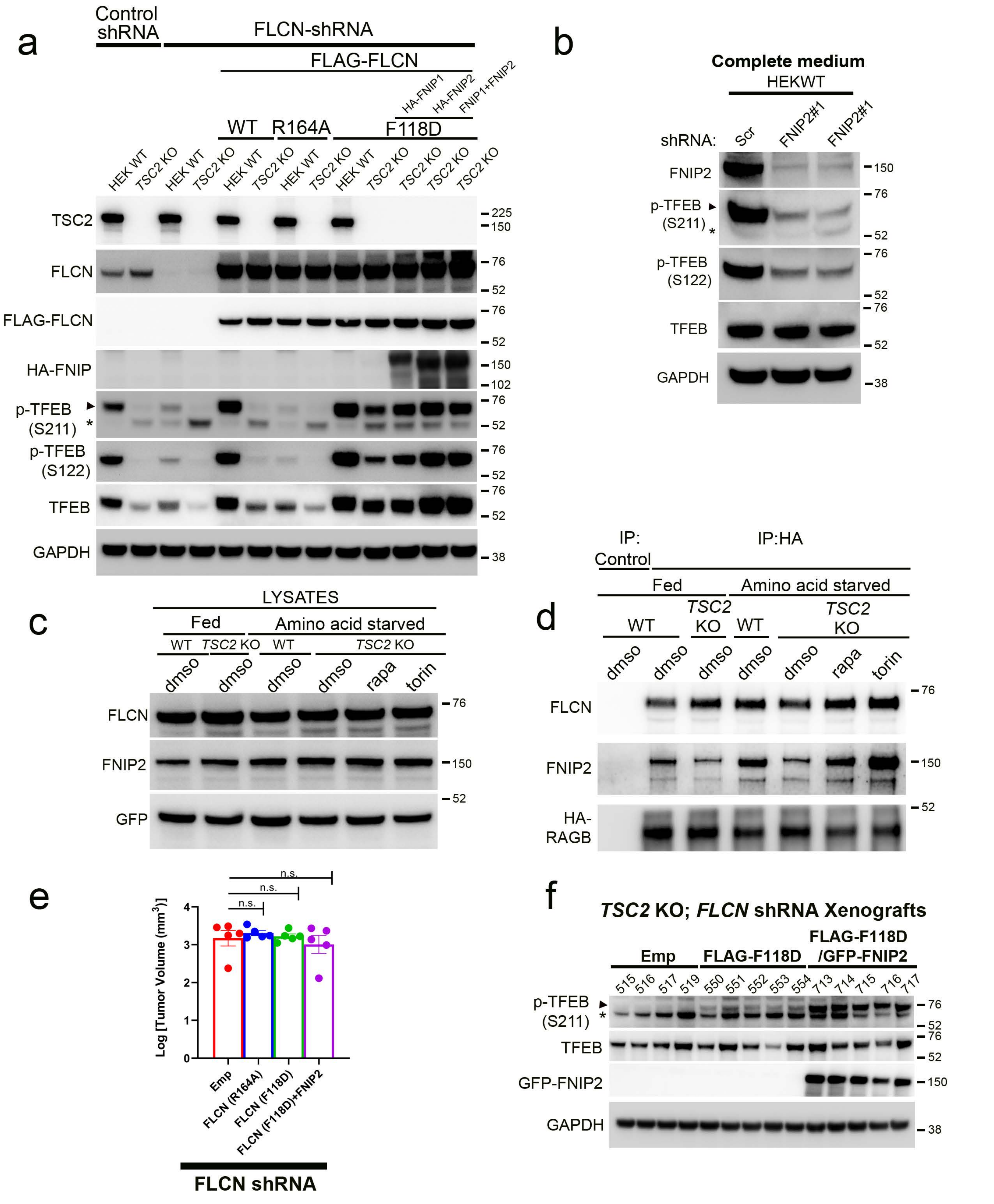

Supplementary Figure S9: ***Restoration of FLCN: FNIP2 lysosomal localization by mTORC1 inhibition or co-expression of FLCN and FNIP2 fully rescues TFEB phosphorylation in cells with TSC2 loss*** **(a)** Immunoblotting of whole cell lysates from WT and *TSC2* KO cells expressing control or *FLCN* shRNA (lanes 1-4) and *FLCN*-depleted WT and *TSC2* KO cells stably expressing FLAG-tagged, WT-FLCN (lanes 5-6), or FLCN mutants with abolished (R164A-FLCN; lanes 7-8) or uninhibited (F118D-FLCN; lanes 9-10) Rag C GAP activity. In the same experiment, *TSC2* KO cells stably expressing F118D-FLCN were transiently transfected with HA-tagged FNIP1, FNIP2 or FNIP1 and FNIP2 (lanes 11-13), prior to lysis and immunoblotting. **(b)** Immunoblotting of WT scrambled control shRNA and *FNIP2* shRNA-transfected cells (two independent target sequences) for FNIP2, p-TFEB and TFEB. **(c)** Immunoblotting of whole cell lysates from immunoprecipitation experiments in Figure 7 b, c. **(d)** WT and *TSC2* KO cells were transiently transfected with a RagB-HA plasmid for 24 hrs, treated with dmso (vehicle), rapamycin (200 nM) or torin1 (1 µM) for 1 hr and maintained in complete growth media or amino acid-starved for 1 hr prior to lysis as indicated, followed by immunoprecipitation (IP) with either a binding control or anti-HA beads coupled to magnetic agarose beads and immunoblotting. **(e)** *In vivo* growth and tumor volume of subcutaneous xenografts in NSG mice derived from *FLCN*-depleted *TSC2* KO cells stably expressing FLAG-tagged R164A-FLCN, F118D-FLCN or F118D-FLCN/FNIP2-GFP. n=5 independent biological replicates. Graphs are presented as mean values $\pm$ SEM. Statistical analyses were performed using one-way ANOVA with Dunnett's test for multiple comparisons. **(f)** Immunoblotting of tumor lysates from *FLCN*-depleted *TSC2* KO xenografts (from

experiments in e) expressing F118D-FLCN or F118D-FLCN/FNIP2-GFP. Source data are provided as a Source Data file.

Supplementary Methods

Cell culture: All cell lines were maintained in DMEM high glucose medium (#11995065, Gibco) with l-glutamine, 10% heat-inactivated FBS (#SH30071.03HI, Hyclone) and 1% penicillin/ streptomycin at 37°C in 5% CO_2. For experiments involving amino acid starvation, cells were rinsed in PBS and incubated in amino acid-free DMEM (#MBS6120661, MyBioSource) supplemented with 10% dialyzed serum (#26400044, Gibco) for 60-90 min. For amino acid addback, cells were stimulated with a 1X mix of essential (#11130036, Thermo Fisher Scientific) and non-essential amino acids (#11140035, Thermo Fisher Scientific) and 200 mM L-Glutamine (#25030081, Thermo Fisher Scientific), added directly to starved cells for 30 min.

Plasmids and Lentiviral transfections: The following plasmids were used: p-EGFP-N1-TFEB (38119, Addgene), pEGFP-N1-delta30-TFEB (44445, Addgene), pEGFP-N1-TFEB-S3A,R4A (44446, Addgene), p-EGFP-N1-TFE3 (38120, Addgene), pRK5-HA GST RagB wt (19301, Addgene), pRK5-HA GST RagB 54L (19302, Addgene), pRK5-HA GST RagB 99L (19303, Addgene), pRK5-HA GST RagC wt (19304, Addgene), pRK5-HA GST RagC 75L (19305, Addgene), pRK5-HA GST RagC 120L (19306, Addgene), pRK5-HA GST RagD 77L (19308, Addgene), [pSK729] pRK5 HA-FLCN (R164A) (136147, Addgene), N1-p18-EGFP (42334, Addgene), FLCN-eGFP (72289, Addgene), pRK5-HA-FNIP1 (72293, Addgene), pRK5-HA-FNIP2 (72295, Addgene), pcDNA3-HA-14-3-3 gamma (13274, Addgene), 4XCLEAR-luciferase reporter (66800, Addgene), pLKO.1 scramble shRNA (1864, Addgene), pLKO.1 FLCN lentiviral shRNA (TRCN0000237886, RNAi Consortium, Sigma), pLKO.1 FNIP2 lentiviral shRNA

(TRCN0000256305; TRCN0000256306, RNAi Consortium, Sigma) and pLKO.1 RRAGD lentiviral shRNA (30321, Addgene). An ORF expression clone for human FNIP2 (NM_020840.3) in a lentiviral vector with N-eGFP and puromycin resistance was custom produced by GeneCopoeia.

Antibodies and Reagents:

Primary antibodies:

TSC1 (6935, Cell Signaling), 1:2000; **TSC2** (4308, Cell Signaling), 1:2000; **Raptor** (2280, Cell Signaling), 1:1000; **Phospho-p70 S6 Kinase (Thr389)** (9205, Cell Signaling), 1:1000; **p70 S6 Kinase** (9202, Cell Signaling), 1:1000; **Phospho-S6 Ribosomal Protein (Ser235/236)** (4858, Cell Signaling), 1:2000; **S6 Ribosomal Protein** (2317, Cell Signaling), 1:2000; **Phospho-4E BP1 (Ser65)** (9451, Cell Signaling), 1:2000; **Phospho-4E BP1 (Thr37/46)** (2855, Cell Signaling), 1:1000; **4E-BP1** (9644, Cell Signaling), 1:2000; **β-Actin** (3700, Cell Signaling), 1:4000; **Gapdh** (2118, Cell Signaling), 1:4000; **Phospho-Akt (S473)** (4060, Cell Signaling), 1:1000; **Akt (pan)** (4691, Cell Signaling), 1:1000; **LAMP-1** (9091, Cell Signaling), 1:2000; **LAMP-2** (ABL-93, DSHB at the University of Iowa), 1:50; **CTSB** (31718, Cell Signaling), 1:1000; **CTSD** (sc-6486, Santa Cruz), 1:500; **CTSK** (ab19027, Abcam), 1:1000; **LC3A/B** (12741, Cell Signaling), 1:1000; **GPNMB** (38313, Cell Signaling), 1:1000; **p62/SQSTM1** (88588, Cell Signaling), 1:1000; **Rab7** (9367, Cell Signaling), 1:1000; **LAMTOR1** (8975, Cell Signaling), 1:1000; **LAMTOR2** (8145, Cell Signaling), 1:1000; **LAMTOR3** (8168, Cell Signaling), 1:1000; **FLCN** (3697, Cell Signaling), 1:1000; ; **FNIP2** (57612, Cell Signaling), 1:1000; **FNIP2** (HPA042779, Sigma), 1:1000; **RagA** (4357, Cell Signaling), 1:1000;

RagB (8150, Cell Signaling), 1:1000; **RagC** (5466, Cell Signaling), 1:1000; **RagD** (4470, Cell Signaling), 1:1000; **GABARAP** (13733, Cell Signaling), 1:1000; **TFEB** (A303-673A, Bethyl), 1:500; **TFE3** (PA5-54909, Thermo Fisher Scientific), 1:500**; TFEB** (4240, Cell Signaling), 1:1000; **TFE3** (14779, Cell Signaling), 1:1000; **Phospho-TFEB (S211)** (37681, Cell Signaling), 1:500; **Phospho-TFEB (S122)** (86843, Cell Signaling), 1:500; **Histone H3** (4499, Cell Signaling), 1:1000; **Fibrillarin** (2639, Cell Signaling), 1:1000; **Lamin A/C** (4777, Cell Signaling), 1:1000; **Ki-67** (12202, Cell Signaling), 1:1000; **GFP** (55494, Cell Signaling), 1:1000; **GFP** (2956, Cell Signaling), 1:1000; **HA** (3724, Cell Signaling), 1:1000; **FLAG** (14793, Cell Signaling), 1:1000; **14-3-3 (pan)** (8312, Cell Signaling), 1:1000.

Reagents: Cell lysis Buffer (9803, Cell Signaling), RIPA buffer (R0278, Sigma), Fibronectin (F1141, Sigma), Rapamycin (R-5000, LC Laboratories), Torin1 (14379, Cell Signaling), Lipofectamine 3000 reagent (L3000001, Thermo Fisher Scientific), Cycloheximide (C4959, Sigma), Chloroquine (C6628, Sigma), Hydroxychloroquine (S4430, Selleck).

Animal Studies:

Genomic DNA was isolated from tail snips and genotyping performed using the following primers:

-Wild-type and floxed *Tsc1*: 5’-GAA TCA ACC CCA CAG AGC AT-3’ (forward)
5’-GTC ACG ACC GTA GGA GAA GC-3’ (reverse)

-Wild-type and floxed *Tsc2*: 5’-ACA ATG GGA GGC ACA TTA CC-3’ (forward)
5’-AAG CAG CAG GTC TGC AGT G-3’ (reverse)

-Wild-type, mutant *Tsc2*: 5'-AAA GCT CAG CCC TTT TCC TC -3' (Wild type forward)

5'-CAA ACC CAC CTC CTC AAG C-3' (Common)

5'-GCC AGA GGC CAC TTG TGT AG-3' (Mutant forward)

-Wild-type, mutant *Pax8cre*: 5'-TGG TAT GTG GTG AAT TTC GTG-3' (Wild type reverse)

5'-GTG GAG GGA CCA CTG AAA GA-3' (Common)

5'-CAG GTT CTT GCG AAC CTC AT-3' (Mutant reverse)

-Wild-type, mutant *ERcre*: 5'-CTG GCT TCT GAG GAC CG-3' (Wild type forward)

5'-CGT GAT CTG CAA CTC CAG TC-3' (Mutant forward)

5'-CCG AAA ATC TGT GGG AAG TC-3' (Wild type reverse)

5'-AGG CAA ATT TTG GTG TAC GG-3' (Mutant reverse)

Xenograft studies: The following groups of cells were used for xenograft studies:

a) HEK293T *TSC2* KO Control, *TFEB* CRISPR, *TFE3* CRISPR and dual *TFEB/TFE3* CRISPR clones.

b) *TSC2* KO *FLCN* shRNA control cells or *TSC2* KO *FLCN* shRNA cells stably expressing $FLCN^{R164A}$, $FLCN^{F118D}$ or $FLCN^{F118D}$+ WT FNIP2-GFP.

3.0 x 10^6 cells were mixed with growth-factor reduced Matrigel (356231, Corning) 1:1 (v/v) in a final volume of 100 ul, and injected subcutaneously into the flanks of 6-week female NSG (NOD *scid* gamma) mice (obtained from Johns Hopkins Research Animal Resources (RAR)). Tumor volume and body weight were measured every 3 days by using calipers and calculated using the formula (length

x width^2 / 2). Mice were euthanized 21 days after inoculation, and tumors were harvested for histological examination and western blotting.

Treatment of WT and *TSC2* KO xenografts with vehicle, rapamycin or torin was initiated once tumors were palpable. Both drugs were dissolved in 100% N-methyl-2-pyrrolidone (#443778, Sigma-Aldrich) at a stock concentration of 6.25 mg/ml, subsequently diluted 1:4 with sterile 50% PEG400 (#06855- Sigma-Aldrich) to a final concentration of 1.25 mg/ml and delivered by i.p. at the indicated dose, once daily. Rapamycin was used at 10 mg/kg as previously described[1]. Torin treatment was performed as described[2], with some modifications: treatment was initiated at 5 mg/kg and was gradually escalated to 10 mg/kg over a period of 2 weeks. For all xenograft experiments, tumor size did not exceed the maximal tumor size/burden permitted by the Johns Hopkins Animal Care and Use Committee (ACUC) (maximum tumor size for a single spontaneous or implanted tumor tumor that is visible without imaging~2 cm in any dimension in adult mice).

<u>Animal care and oversight:</u> Animal protocols were approved by the JHU Animal Care and Use Committee, under the following protocols:

1) The role of the PI3K/mTOR signaling pathways in murine epithelial morphogenesis and migration (MO20M83)

2) Targeting Lysosomal Biogenesis in Renal Tumors with TSC1/2 Loss (MO20M185).

Animals will have access to food (standard rodent chow) and water ad libitum. Light will be regulated by timer, with 12 hour on/off cycles. Rooms will be maintained at standard mouse temperature (68-79° F) and humidity (30-70% relative humidity).

Laser Capture Microdissection: Frozen, O.C.T.-embedded kidney sections from *Tsc2 +/-* A/J mice were prepared for LCM processing as follows: tissues were cut in 10-μM thick sections on 4 μM polyethylene naphthalate (PEN) membrane slides (#11600288; Leica) for laser capture microdissection (LCM) and 4 μM each on a regular slide for hematoxylin and eosin staining (H&E). Before use for sectioning, PEN slides were sterilized overnight under ultra violet radiation to remove RNAses. Sections were stained with Hematoxylin and LCM was performed using a Leica DM7000 microscope. Tumor regions were isolated and stored in Buffer RLT lysis buffer (Qiagen). Respective H&E/ p-S6-stained sections were used as a reference to identify the morphology of the tissue and renal tumors. LCM sorted cells were then homogenized using QIAshredder (QIAGEN) and total RNA was extracted using the RNeasy kit (QIAGEN). qRT-PCR was performed as described below.

Histology and immunostaining: Mouse kidneys were fixed in 10% neutral buffered formalin (Sigma-Aldrich), embedded in paraffin, sectioned at 4 μm and used for immunohistochemistry. Immunostaining of renal cystadenoma tissues from *Tsc2* +/- mice with a) p-S6, b) CTSB, c) Lamtor 1, 4) LC3 A, B, 5) RagC, 6) Ki-67 and 7) LAMP1 was performed using a manual staining protocol as follows: Sections were deparaffinized in xylene (Sigma-Aldrich), hydrated in graded ethanol and rinsed in distilled water. Antigen retrieval was performed using citrate (10 mM, pH 6.0) or EDTA + 0.01% TWEEN 20 (1 mM, pH 8.0) buffers and HIER (heat-induced epitope retrieval) method, in accordance with the protocol specified for each antibody. All washing steps were done using 1X TBS-T buffer. Endogenous peroxidase activity was quenched by incubation with Dual Enzyme

Block (Dako, Agilent Technologies) for 10 minutes at room temperature. Sections were incubated with each antibody overnight at 4°C diluted in antibody dilution buffer (Roche/ Ventana Medical Systems). For immunohistochemistry, a horseradish peroxidase–labeled polymer, Poly-HRP PowerVision Detection System (Novocastra/Leica Biosystems) was applied for 30 minutes at room temperature. Signal detection was performed using 3,3'-diaminobenzidine tetrahydrochloride (DAB) (Sigma-Aldrich) for 20 minutes at room temperature. Slides were counterstained for 30 seconds with Mayer's hematoxylin (Dako, Agilent Technologies), dehydrated, and mounted. For immunofluorescence, after primary antibody overnight reaction at 4°C, sections were incubated with secondary antibodies (Alexafluor-488 or Alexafluor-594 conjugated, anti-Rabbit or anti-Mouse IgG, Thermo Fisher Scientific) at a dilution of 1:200 for 1h at room temperature. Subsequently they were washed 2x/5min in PBS, rinsed in distilled water, dehydrated in graded ethanol and mounted with ProLong Gold Antifade with DAPI (Thermo Fisher Scientific).

TFEB, TFE3, TSC2 and FNIP2 Immunohistochemistry (IHC) on human, murine and xenograft tissues was performed on the Ventana Discovery ULTRA (version v12.31) (Ventana/ Roche) using hand-applied TFEB (A303-673A, Bethyl-murine tissues), 1:1000; TFEB (4240, Cell Signaling-human and xenograft tissues), 1:200; TFE3 (PA5-54909, Thermo Fisher Scientific-all tissues), 1:5000; TSC2 (4308, Cell Signaling), 1:100 and FNIP2 (HPA042779, Sigma-murine tissues), 1:100.

Quantification of nuclear TFE3/B in murine renal tumors: Immunostained slides were digitally scanned (Nanozoomer, Hamamatsu). Cell nuclei were identified with the QuPath (0.3.0) “Cell Detection” tool with the brightness and contrast set to the optimal density sum setting. Nuclear intensity was quantified with a three-threshold setting using the tool “set cell intensity classifications” and using the channel “DAB OD Max”. Percent positive, H-score, and cell number at each threshold was analyzed. Identical analysis was done for only visually identified tumors which was then subtracted from whole tissue area.

Quantification of nuclear TFE3/B in xenografts: Immunostained slides were digitally scanned (Nanozoomer, Hamamatsu). Tissue was stained with anti-TFE3/B antibody in brown. Blood vessels were manually identified following which, “expand annotations” was used to create two expansion areas, each with a 60 micron radius. Cell nuclei were identified with the QuPath (0.3.0) “Cell Detection” tool, with the brightness and contrast set to the optimal density sum setting, and the minimum necessary nuclei size zero. A 2.5-micron expansion from the nuclei was made to create a cytoplasmic area. Necrotic regions were removed by setting a threshold for “Cell: DAB OD min” under “create single measurement classifier” tool. Intensity of all nuclei was quantified with a three-threshold setting using the tool “set cell intensity classifications” and using the channel “DAB OD Max”. Percent positive, H-score, and cell number at each threshold and each expansion (0-60 microns and 60-120 microns) was analyzed.

Cell and tissue lysates, immunoblotting and immunoprecipitation: Tumor xenografts were homogenized and lysed using the gentleMACS M Tubes/ Octo Dissociator in ice-

cold cell lysis buffer (#9803, Cell Signaling). Cells were lysed in RIPA buffer (for lysosomal protein expression) or cell lysis buffer (#9803, Cell Signaling) (for all other experiments and immunoprecipitations). All lysis buffers were supplemented with $NaVO_4$ (1 mM), NaF (1 mM) and 10 µl Halt Protease and Phosphatase Inhibitor Cocktail (#78440, Thermo Fisher Scientific). Lysates were centrifuged at 21,000 rpm for 10 minutes at 4°C and supernatants collected. Protein concentrations were quantified using the BCA Protein Assay Kit (#23225, Pierce), and protein was resolved on 4-12% Bis-Tris SDS-PAGE gel (Thermo Fisher Scientific). Protein was transferred to nitrocellulose membranes (Amersham Bioscience), blocked for 1h at room temperature in 5% nonfat milk in 1X TBS-T and then incubated overnight with a primary antibody diluted in 5% BSA or milk in 1X TBS-T. The secondary antibodies used were anti-rabbit or anti-mouse immunoglobulin as appropriate (Cell Signaling) and diluted at 1:1000 in 5% nonfat milk in 1X TBS-T. Blots were developed using a chemiluminescent development solution (Super Signal West Femto, Pierce) and bands were imaged on a chemiluminescent imaging system (ChemiDoc Touch imaging System using the ImageLab Touch Software (version 2.3.0.07) (Bio-Rad) or MicroChemi Chemiluminescent imager using the GelCapture Software (version 2.2.2.0) (FroggaBio Inc.). Digital images were quantified using Image J (version 1.52p) and all bands were normalized to their respective β-actin or GAPDH expression levels as loading controls. Immunoprecipitation of TFEB-GFP, TFE3-GFP and Lamor1-GFP was performed using anti-GFP trap magnetic agarose beads (GTMA-20, Chromotek) as per manufacturer instructions. Immunoprecipitation of RagB-HA and HA-14-3-3 gamma was performed using Pierce Anti-HA Magnetic Beads (88337, Thermo Fisher Scientific) as per manufacturer instructions.

Nuclear lysates were prepared using the PARIS kit (AM1921, Thermo Fisher Scientific) according to manufacturer's instructions. Digital images were quantified using Image J and all bands were normalized to their respective Lamin, Histone H3 or Fibrillarin levels as loading controls. Statistical analysis was performed using Student's unpaired t-test or one-way ANOVA.

Lysosomal fractionation assays: Lysosomal fractionation assays were carried out as previously described [3]. Cells grown on 150 mm dishes were harvested and lysed in 750 mL of cold fractionation buffer (50 mM KCl, 90 mM potassium gluconate, 1 mM EGTA, 50 mM sucrose, 5 mM glucose, protease inhibitor cocktail tablet, and 20 mM HEPES, pH 7.4). The cells were then lysed by syringing, and nuclear fraction was removed by centrifugation at 1000*g* for 10 minutes at 4°C. The supernatant was then centrifuged at 20,000*g* for 30 minutes at 4°C. The precipitated lysosome-enriched fraction (LEF) was re-suspended in the fractionation buffer, and the supernatant was separated as the cytosolic fraction.

Luciferase reporter assays: HEK293T and *TSC2* KO cells were co-transfected with 500 ng of the 4XCLEAR-luciferase reporter plasmid and 1 ng of a Renilla luciferase vector using Lipofectamine 3000 reagent for 48 hrs. Cells were lysed with 1X Passive Lysis Buffer and luciferase assay performed using the Dual-Luciferase Reporter Assay system (E1910, Promega) on a GloMax Multi Detection System with Instinct Software (version 3.1.2) (Promega) according to manufacturer guidelines.

RNA isolation and quantitative real-time RT-PCR: Total cellular RNA was extracted using RNeasy Mini kit (#74104, Qiagen) according to manufacturer's instructions. RNA was converted to cDNA using SuperScript III First-Strand Synthesis System (#18080051, Thermo Fisher Scientific) according to manufacturer's instructions. mRNA levels were quantified using an ABI Prism 7900HT Real-time PCR system using the SDS (Sequence Detection System) software (version 2.4) (Applied Biosystems) with the following human primers and probes: **{CTSK** (Hs00166156_m1), **RRAGD** (Hs00222001_g1, **SQSTM1** (Hs01061917_g1), **UVRAG (**Hs01075434_m1), **WIPI1 (**Hs00924447_m1), **FLCN (**Hs00376065_m1), **PPARG (**Hs01115513_m1), **GPNMB (**Hs01095669_m1) and **GAPDH (**Hs02786624_g1)**},** and mouse primers and probes: **{LAMP1** (Mm00495262_m1), **CTSB** (Mm01310506_m1), **CTSD** (Mm00515586_m1), **MCOLN1** (Mm00522550_m1), **ATP6AP2** (Mm00510396_m1), **SQSTM1** (Mm00448091_m1) and **ACTB** (Mm02619850_m1)**}**. Threshold cycle (Ct) was obtained from the PCR reaction curves and mRNA levels were quantitated using the comparative Ct method with actin or Gapdh mRNA serving as the reference. Statistical analysis was performed using Student's unpaired t-test.

Immunocytochemistry: HEK293T cells seeded on fibronectin-coated coverslips were either fixed in 100% methanol at -20°C for 30 minutes or 4% PFA for 15 minutes at room temperature, according to antibody specifications. Following three rinses in 1X PBS, cells were permeabilized and blocked in a buffer containing 1X PBS, 5% normal donkey serum and 0.3% Triton X-100. For immunofluorescence, coverslips were incubated with the indicated primary antibodies overnight at 4°C in antibody dilution buffer (ADB) containing

1X PBS, 1% BSA and 0.3% Triton X-100. After 3 rinses of 1X PBS, coverslips were incubated with secondary antibodies (Alexafluor-488 or Alexafluor-594 conjugated, anti-Rabbit or anti-Mouse IgG, Thermo Fisher Scientific) in ADB at a dilution of 1:200 for 1 hour at room temperature. Nuclei were counterstained with DAPI and coverslips visualized using an Olympus BX41 epifluorescence microscope using DP Controller software (version 3.2.1.276) (Olympus, Center Valley, PA).

Immunofluorescence image analysis and quantification

Nuclear TFEB-GFP and TFE3-GFP: Image analysis and quantification was done in CellProfiler (version 4.2.4). Cells were stained with DAPI to mark nuclei (blue channel) and anti-GFP (red channel). All images were captured using the same exposure for each stain within each biological replicate. The blue channel was separated and converted to 16-bit grayscale to be thresholded to a binary image. Nuclei were identified using the "Identify Primary Objects" function. Red channel was converted to a 16-bit grayscale image and then thresholded to a binary image and overlaid with nuclei to isolate only nuclei of cells with anti-GFP staining – all other nuclei were discarded. New red and blue channels were re-merged to identify whole cells with GFP staining by thresholding to a binary image and identified with the function "Identify Secondary Objects." Nuclei were subtracted from whole cells using the function "Identify Tertiary Objects" to identify cytoplasm. GFP-stained nuclear and cytoplasmic mean area intensity was separately measured and divided on a cell-by-cell basis to produce whole image, nuclear-to-cytoplasmic GFP-TFE3 or GFP-TFEB mean intensity.

Endogenous nuclear TFEB and TFE3 quantification: Cells were stained with DAPI to mark nuclei (blue channel) and anti-total TFEB/TFE3 (red/green channel). Images were analyzed using ImageJ. The blue channel was used to segment nuclei as follows: images were thresholded to remove background and converted to binary images, following which the analyze particles function was used for automatic detection of nuclear outlines. These nuclear outlines were applied to the red channel and mean fluorescence intensity of TFEB/TFE3 within the regions was measured.

Statistics for image analysis: Normal distribution was assessed using the D'Agostino & Pearson normality test. If normally distributed, statistical significance was determined with Student's t-test when comparing two experimental groups, or with one-way ANOVA with Dunnett's or Bonferroni's correction when comparing 3 or more experimental groups. If not normally distributed, statistical significance was determined with the Mann-Whitney test when comparing two experimental groups, or with the Kurskal-Wallis test with Dunn's correction when comparing 3 or more experimental groups. All tests assumed a two-tailed deviation and were performed in GraphPad Prism (version 8.2.1).

Cathepsin D activity assays: Cathepsin D enzyme activity was determined using a Cathepsin D activity assay kit (ab65302, Abcam), a fluorescence-based assay that utilizes the preferred cathepsin-D substrate sequence GKPILFFRLK(Dnp)-D-R-NH2 labeled with MCA, according to manufacturer's instructions. Fluorescent microplate measurements were obtained using GloMax Multi Detection System with Instinct Software (version 3.1.2) (Promega).

Confluence assays: IncuCyte ZOOM live-cell imaging system (Essen Bioscience, Ann Arbor, MI) was used for kinetic monitoring of cell proliferation in *TSC2* KO control and *TFEB/TFE3* KO cells. Cells were seeded at a density of 20,000 cells/ well of a 48 well plate in complete media. The plate was scanned and phase contrast images were acquired in real time every 4 hrs from 0 to 48 hrs, using a ZOOM 4× objective (Nikon Plan Apo Lambda 4×/0.20; cat. no. 4466). Cell confluence (%) was calculated and analyzed by the IncuCyte ZOOM integrated software (version 2016B) (Essen Bioscience).

Cell Viability assays: Cell viability was analyzed using a colorimetry-based assay using the CellTiter 96® Aqueous One Solution Cell Proliferation Assay (MTS) (G3582, Promega), according to manufacturer's instructions. Colorimetric measurements were obtained using the xMark Microplate Spectrophotometer and Microplate Manager Software (version 6.3) (Biorad).

References:

1. Totary-Jain, H. *et al.* Rapamycin resistance is linked to defective regulation of Skp2. *Cancer Res* **72**, 1836-1843 (2012).
2. Liu, Q. *et al.* Discovery of 1-(4-(4-propionylpiperazin-1-yl)-3-(trifluoromethyl)phenyl)-9-(quinolin-3-yl)benzo[h][1,6]naphthyridin-2(1H)-one as a highly potent, selective mammalian target of rapamycin (mTOR) inhibitor for the treatment of cancer. *J Med Chem* **53**, 7146-7155 (2010).
3. Kim, Y.C. *et al.* Rag GTPases are cardioprotective by regulating lysosomal function. *Nat Commun* **5**, 4241 (2014).
