# Supplementary material for: An mTORC1-mediated negative feedback loop constrains amino acid-induced FLCN-Rag activation in renal cells with TSC2 loss
Source: Nat Commun. 2022 Nov 10;13:6808. doi: 10.1038/s41467-022-34617-7 (PMC9649702; doi:10.1038/s41467-022-34617-7)
Supplement: Supplementary file 3 — Reporting Summary [file 41467_2022_34617_MOESM3_ESM.pdf]

Corresponding author(s): Tamara Lotan (T.L.L.) (tlotan1@jhmi.edu)

Last updated by author(s): Oct 26, 2022

Reporting Summary

Nature Portfolio wishes to improve the reproducibility of the work that we publish. This form provides structure for consistency and transparency in reporting. For further information on Nature Portfolio policies, see our Editorial Policies and the Editorial Policy Checklist.

Statistics

For all statistical analyses, confirm that the following items are present in the figure legend, table legend, main text, or Methods section.

n/a	Confirmed	
☐	☒	The exact sample size (*n*) for each experimental group/condition, given as a discrete number and unit of measurement
☐	☒	A statement on whether measurements were taken from distinct samples or whether the same sample was measured repeatedly
☐	☒	The statistical test(s) used AND whether they are one- or two-sided<br>*Only common tests should be described solely by name; describe more complex techniques in the Methods section.*
☒	☐	A description of all covariates tested
☐	☒	A description of any assumptions or corrections, such as tests of normality and adjustment for multiple comparisons
☐	☒	A full description of the statistical parameters including central tendency (e.g. means) or other basic estimates (e.g. regression coefficient) AND variation (e.g. standard deviation) or associated estimates of uncertainty (e.g. confidence intervals)
☐	☒	For null hypothesis testing, the test statistic (e.g. *F*, *t*, *r*) with confidence intervals, effect sizes, degrees of freedom and *P* value noted<br>*Give P values as exact values whenever suitable.*
☒	☐	For Bayesian analysis, information on the choice of priors and Markov chain Monte Carlo settings
☒	☐	For hierarchical and complex designs, identification of the appropriate level for tests and full reporting of outcomes
☒	☐	Estimates of effect sizes (e.g. Cohen's *d*, Pearson's *r*), indicating how they were calculated

Our web collection on statistics for biologists contains articles on many of the points above.

Software and code

Policy information about availability of computer code

Data collection

1) All immuno-blotting data were imaged on a chemiluminescent imaging system :
 a) ChemiDoc Touch Imaging System using the ImageLab Touch Software (version 2.3.0.07) (Biorad) or,
 b) Gel Capture Micro Chemi Unit and GelCapture Software (version 2.2.2.0) (FroggaBio Inc.)
2) All mRNA levels were quantified using an ABI Prism 7900HT Real-time PCR system using the SDS (Sequence Detection System) software (version 2.4) (Applied Biosystems).
3) All immunofluorescence images were visualized using an Olympus BX41 epifluorescence microscope using DP Controller software (version 3.2.1.276) (Olympus, Center Valley, PA).
4) All measurements for luciferase reporter assays and fluorescence microplate assays (Cathepsin D activity assay) were obtained using GloMax Multi Detection System with Instinct Software (version 3.1.2) (Promega).
5). IncuCyte ZOOM live-cell imaging system (Essen Bioscience, Ann Arbor, MI) was used for kinetic monitoring of cell proliferation.
6) Laser Capture Microdissection was performed using a Leica DM7000 microscope.
7) Immunohistochemistry (IHC) on human, murine and xenograft tissues was performed on the Ventana Discovery ULTRA (version v12.31) (Ventana/ Roche).
8) All colorimetry-based protein estimations and MTS assays were performed using the xMark Microplate Spectrophotometer and Microplate Manager Software (version 6.3) (Biorad).

Data analysis	1) All immunoblots were quantified using Image J (version 1.52p).<br>2) Nuclear TFEB and TFE3 fluorescent image analysis and quantification was done using FIJI (Image J 1.52g) or CellProfiler (version 4.2.4)<br>3) Quantification of nuclear TFEB and TFE3 in murine renal tumors and xenografts was performed on digitally scanned immunostained slides (Nanozoomer, Hamamatsu), using QuPath (0.3.0).<br>4) Cell confluence (%) was calculated and analyzed by the IncuCyte ZOOM integrated software (version 2016B)(Essen Bioscience).<br>5) All statistical analyses were performed using GraphPad Prism (version 8.2.1)

For manuscripts utilizing custom algorithms or software that are central to the research but not yet described in published literature, software must be made available to editors and reviewers. We strongly encourage code deposition in a community repository (e.g. GitHub). See the Nature Portfolio guidelines for submitting code & software for further information.

Data

Policy information about availability of data

All manuscripts must include a data availability statement. This statement should provide the following information, where applicable:

- Accession codes, unique identifiers, or web links for publicly available datasets
- A description of any restrictions on data availability
- For clinical datasets or third party data, please ensure that the statement adheres to our policy

Data availability:
All data generated and analyzed during the current study are included in this published article and its supplementary information files, or are deposited in GEO. All unique materials generated (such as the TFEB/TFE3 KO CRISPR cell lines) during this study are available from the corresponding author upon request. A reporting summary for this article is available as a Supplementary information file. Source data for Figs.1a, c-e, g, i, j, 2a, b, d-h, j-l, 3b, 4a-b, 5a-e, 6a-f, 7a-f, and Suppl Figs. 1a,c,d 2d-g, 3b, 4a-b, 6a-d, 7a-f, 8a-c, 9a-f are provided with this paper in a Source data file with a separate supplementary pdf file with uncropped western blots. Literature-curated lysosomal gene sets from the following studies were used for Gene Set Enrichment Analysis: 1) The Human Lysosome Gene Database (hLGDB) (Brozzi et al. 2013; https://pubmed.ncbi.nlm.nih.gov/23584836/), 2) Sardiello et al. (2009; https://pubmed.ncbi.nlm.nih.gov/19556463/) (GSE16267), 3) Perera et al. (2015; https://pubmed.ncbi.nlm.nih.gov/26168401/) (GSE62077) and Hoek et al. (2008; https://pubmed.ncbi.nlm.nih.gov/19067971/). The RNA-seq data from this study are deposited into NCBI's Gene Expression Omnibus (GEO) database with the accession code GSE216545 (https://www.ncbi.nlm.nih.gov/geo/query/acc.cgi?acc=GSE216545). Source data are provided with this paper.

Field-specific reporting

Please select the one below that is the best fit for your research. If you are not sure, read the appropriate sections before making your selection.

☒ Life sciences ☐ Behavioural & social sciences ☐ Ecological, evolutionary & environmental sciences

For a reference copy of the document with all sections, see nature.com/documents/nr-reporting-summary-flat.pdf

Life sciences study design

All studies must disclose on these points even when the disclosure is negative.

Sample size	Sample sizes for xenograft experiments were not predetermined as experiments were exploratory in nature and multiple clones were tested for each genotype. For all other experiments, appropriate sample sizes were determined based on the variability of the experimental assay, with a minimun n=3 for all experiments.
Data exclusions	No data were excluded from the analyses.
Replication	All experiments were replicated in three or more independent biological replicates. Additionally, multiple orthogonal techniques were utilized to ensure rigor. For example: a) TFEB/TFE3 nuclear localization was confirmed by immunofluorescence (cells), IHC (mouse renal tumors and xenografts) and immunoblotting of nuclear-cytoplasmic fractions, b) increased TFEB/TFE3 transcriptional activity was confirmed by qRT-PCR, immunoblotting of lysosomal proteins, RNASeq and 4XCLEAR promoter activity assay.
Randomization	For xenograft experiments with drug treatments, once tumors developed, NSG mice were randomly assigned to vehicle or drug treatement groups. For all other experiments, randomization was not relevant.
Blinding	Due to limited number of personnel able to perform and/or analyze assays, investigators were not blinded during data collection and/or analyses. Immunofluorescence, IHC, and immunoblotting experiments were analyzed using automated, software-based quantification methods (CellProfiler/ QuPath/Image J), and quantification parameters were applied equally to all samples and replicates to reduce bias.

Reporting for specific materials, systems and methods

We require information from authors about some types of materials, experimental systems and methods used in many studies. Here, indicate whether each material, system or method listed is relevant to your study. If you are not sure if a list item applies to your research, read the appropriate section before selecting a response.

Materials & experimental systems

n/a	Involved in the study
☐	☒ Antibodies
☐	☒ Eukaryotic cell lines
☒	☐ Palaeontology and archaeology
☐	☒ Animals and other organisms
☒	☐ Human research participants
☒	☐ Clinical data
☒	☐ Dual use research of concern

Methods

n/a	Involved in the study
☒	☐ ChIP-seq
☒	☐ Flow cytometry
☒	☐ MRI-based neuroimaging

Antibodies

Antibodies used

Primary antibodies:
TSC1 (6935, Cell Signaling), 1:2000; TSC2 (4308, Cell Signaling), 1:2000; Raptor (2280, Cell Signaling), 1:1000; Phospho-p70 S6 Kinase (Thr389) (9205, Cell Signaling), 1:1000; p70 S6 Kinase (9202, Cell Signaling), 1:1000; Phospho-S6 Ribosomal Protein (Ser235/236) (4858, Cell Signaling), 1:2000; S6 Ribosomal Protein (2317, Cell Signaling), 1:2000; Phospho-4E BP1 (Ser65) (9451, Cell Signaling), 1:2000; Phospho-4E BP1 (Thr37/46) (2855, Cell Signaling), 1:1000; 4E-BP1 (9644, Cell Signaling), 1:2000; Phospho-ULK1 (Ser757) (6888, Cell Signaling), 1:2000; ULK1 (8054, Cell Signaling), 1:2000; β-Actin (3700, Cell Signaling), 1:4000; Gapdh (2118, Cell Signaling), 1:4000; Phospho-Akt (S473) (4060, Cell Signaling), 1:1000; Akt (pan) (4691, Cell Signaling), 1:1000; LAMP-1 (9091, Cell Signaling), 1:2000; LAMP-2 (ABL-93, DSHB at the University of Iowa), 1:50; CTSB (31718, Cell Signaling), 1:1000; CTSD (sc-6486, Santa Cruz), 1:500; CTSK (ab19027, Abcam), 1:1000; ATP6V0A1 (sc-374475, Santa Cruz), 1:500; LC3A/B (12741, Cell Signaling), 1:1000; GPNMB (38313, Cell Signaling), 1:1000; p62/SQSTM1 (23214, Cell Signaling), 1:1000; Rab7 (9367, Cell Signaling), 1:1000; LAMTOR1 (8975, Cell Signaling), 1:1000; LAMTOR2 (8145, Cell Signaling), 1:1000; LAMTOR3 (8168, Cell Signaling), 1:1000; FLCN (3697, Cell Signaling), 1:1000; ; FNIP2 (57612, Cell Signaling), 1:1000; FNIP2 (HPA042779, Sigma), 1:1000; RagA (4357, Cell Signaling), 1:1000; RagB (8150, Cell Signaling), 1:1000; RagC (5466, Cell Signaling), 1:1000; RagD (4470, Cell Signaling), 1:1000; GABARAP (13733, Cell Signaling), 1:1000; TFEB (A303-673A, Bethyl), 1:500; TFE3 (PA5-54909, Thermo Fisher Scientific), 1:500; TFEB (4240, Cell Signaling), 1:1000; TFE3 (14779, Cell Signaling), 1:1000; Phospho-TFEB (S211) (37681, Cell Signaling), 1:500; Phospho-TFEB (S122) (86843, Cell Signaling), 1:500; Histone H3 (4499, Cell Signaling), 1:1000; Fibrillarin (2639, Cell Signaling), 1:1000; Lamin A/C (4777, Cell Signaling), 1:1000; Ki-67 (12202, Cell Signaling), 1:1000; GFP (55494, Cell Signaling), 1:1000; GFP (2956, Cell Signaling), 1:1000; HA (3724, Cell Signaling), 1:1000; FLAG (14793, Cell Signaling), 1:1000; 14-3-3 (pan) (8312, Cell Signaling), 1:1000.

TFEB, TFE3, TSC2 and FNIP2 Immunohistochemistry (IHC) on human, murine and xenograft tissues was performed on the Ventana Discovery ULTRA (version v12.31) (Ventana/ Roche) using hand-applied TFEB (A303-673A, Bethyl-murine tissues), 1:1000; TFEB (4240, Cell Signaling-human and xenograft tissues), 1:200; TFE3 (PA5-54909, Thermo Fisher Scientific-all tissues), 1:5000; TSC2 (4308, Cell Signaling), 1:100 and FNIP2 (HPA042779, Sigma-murine tissues), 1:100.

Validation

Primary antibodies:
TSC1 (6935, Cell Signaling)
https://www.cellsignal.com/products/primary-antibodies/hamartin-tsc1-d43e2-rabbit-mab/6935
The antibody was validated by the manufacturer for Western blotting and Immunoprecipitation in humans and mice.
Validation from our study: the antibody detects a band at the expected size on western blots which is completely absent in human HEK293T cells with genomic deletion of TSC1 via CRISPR-Cas9.

TSC2 (4308, Cell Signaling)
https://www.cellsignal.com/products/primary-antibodies/tuberin-tsc2-d93f12-xp-rabbit-mab/4308
The antibody was validated by the manufacturer for Western blotting, Immunofluorescence, Immunoprecipitation and Flow cytometry in humans and mice.
Validation from our study: the antibody detects a band at the expected size on western blots which is completely absent in human HEK293T cells with genomic deletion of TSC2 via CRISPR-Cas9, TSC2 deficient human TRI-102 cells and in murine Tsc2-null TTJ cells (a cell line derived from Tsc2+/- C57BL/6 mice). We additionally validated this antibody by IHC: previously, we confirmed Tsc2 protein loss in renal cystadenomas and tumors in Tsc2 +/- A/J mice using a novel immunohistochemistry assay (PMID: 35072947), consistent with bi-allelic inactivation of Tsc2. In this manuscript, we examined expression of Tsc2 by immunohistochemistry in PAX8-Cre; Tsc2 fl/wt mice. By 18 months of age, renal cystadenomas and cystadenocarcinomas are evident in these mice, with accompanying Tsc2 protein loss (confirming bi-allelic Tsc2 inactivation).

Raptor (2280, Cell Signaling)
https://www.cellsignal.com/products/primary-antibodies/raptor-24c12-rabbit-mab/2280
The antibody was validated by the manufacturer for Western blotting and Immunoprecipitation in humans and mice.
Validation from our study: the antibody detects a band at the expected size on western blots.

Phospho-p70 S6 Kinase (Thr389) (9205, Cell Signaling)
https://www.cellsignal.com/products/primary-antibodies/phospho-p70-s6-kinase-thr389-antibody/9205
The antibody was validated by the manufacturer for Western blotting in humans and mice.
Validation from our study: the antibody detects a band at the expected size on western blots. Additionally, band intensity is increased in TSC2 KO cells, and decreased in response to treatment with rapamycin or torin, consistent with an increase and decrease in mTORC1 activation, respectively.

p70 S6 Kinase (9202, Cell Signaling)

https://www.cellsignal.com/products/primary-antibodies/p70-s6-kinase-antibody/9202
The antibody was validated by the manufacturer for Western blotting and Immunoprecipitation in humans and mice.
Validation from our study: the antibody detects a band at the expected size on western blots.

Phospho-S6 Ribosomal Protein (Ser235/236) (4858, Cell Signaling)
https://www.cellsignal.com/products/primary-antibodies/phospho-s6-ribosomal-protein-ser235-236-d57-2-2e-xp-rabbit-mab/4858
The antibody was validated by the manufacturer for Western blotting, Immunofluorescence, Immunohistochemistry and Flow Cytometry in humans and mice.
Validation from our study: the antibody detects a band at the expected size on western blots. Additionally, band intensity is increased in TSC2 KO cells, and decreased in response to treatment with rapamycin or torin, consistent with an increase and decrease in mTORC1 activation, respectively. Increased staining intensity was also observed in renal cystadenomas and tumors in Tsc2 +/- A/J mice by immunohistochemistry, consistent with increased mTORC1 activity in these tumors.

S6 Ribosomal Protein (2317, Cell Signaling)
https://www.cellsignal.com/products/primary-antibodies/s6-ribosomal-protein-54d2-mouse-mab/2317
The antibody was validated by the manufacturer for Western blotting, Immunofluorescence, Immunohistochemistry and Flow Cytometry in humans and mice.
Validation from our study: the antibody detects a band at the expected size on western blots.

Phospho-4E BP1 (Ser65) (9451, Cell Signaling)
https://www.cellsignal.com/products/primary-antibodies/phospho-4e-bp1-ser65-antibody/9451
The antibody was validated by the manufacturer for Western blotting and Immunoprecipitation in humans and mice.
Validation from our study: the antibody detects a band at the expected size on western blots. Additionally, band intensity is increased in TSC2 KO cells, and decreased in response to treatment with rapamycin or torin, consistent with an increase and decrease in mTORC1 activation, respectively.

Phospho-4E BP1 (Thr37/46) (2855, Cell Signaling)
https://www.cellsignal.com/products/primary-antibodies/phospho-4e-bp1-thr37-46-236b4-rabbit-mab/2855
The antibody was validated by the manufacturer for Western blotting, Immunofluorescence, Immunohistochemistry and Flow Cytometry in humans and mice.
Validation from our study: the antibody detects a band at the expected size on western blots.

4E-BP1 (9644, Cell Signaling)
https://www.cellsignal.com/products/primary-antibodies/4e-bp1-53h11-rabbit-mab/9644
The antibody was validated by the manufacturer for Western blotting, Immunofluorescence, Immunohistochemistry, Flow Cytometry and Immunoprecipitation in humans and mice.
Validation from our study: the antibody detects a band at the expected size on western blots.

β-Actin (3700, Cell Signaling)
https://www.cellsignal.com/products/primary-antibodies/b-actin-8h10d10-mouse-mab/3700
The antibody was validated by the manufacturer for Western blotting, Immunofluorescence, Immunohistochemistry and Flow Cytometry in humans and mice.
Validation from our study: the antibody detects a band at the expected size on western blots.

Gapdh (2118, Cell Signaling)
https://www.cellsignal.com/products/primary-antibodies/gapdh-14c10-rabbit-mab/2118
The antibody was validated by the manufacturer for Western blotting, Immunofluorescence, Immunohistochemistry and Flow Cytometry in humans and mice.
Validation from our study: the antibody detects a band at the expected size on western blots.

Phospho-Akt (S473) (4060, Cell Signaling)
https://www.cellsignal.com/products/primary-antibodies/phospho-akt-ser473-d9e-xp-rabbit-mab/4060
The antibody was validated by the manufacturer for Western blotting, Immunofluorescence, Immunohistochemistry, Flow Cytometry and Immunoprecipitation in humans and mice.
Validation from our study: the antibody detects a band at the expected size on western blots. Additionally, band intensity is decreased in response to treatment with torin, consistent with a decrease in mTORC2 activation.

Akt (pan) (4691, Cell Signaling)
https://www.cellsignal.com/products/primary-antibodies/akt-pan-c67e7-rabbit-mab/4691
The antibody was validated by the manufacturer for Western blotting, Immunofluorescence, Immunohistochemistry, Flow Cytometry and Immunoprecipitation in humans and mice.
Validation from our study: the antibody detects a band at the expected size on western blots.

LAMP-1 (9091, Cell Signaling)
https://www.cellsignal.com/products/primary-antibodies/lamp1-d2d11-xp-rabbit-mab/9091
The antibody was validated by the manufacturer for Western blotting, Immunofluorescence, Immunohistochemistry, Flow Cytometry and Immunoprecipitation in humans.
Validation from our study: the antibody detects a band at the expected size on western blots, in whole cell lysates and lysosomal-enriched fractions.

LAMP-2 (ABL-93, DSHB at the University of Iowa)
https://dshb.biology.uiowa.edu/ABL-93
The antibody was validated by the manufacturer for Western blotting, Immunofluorescence, Immunohistochemistry, FACS and Immunoprecipitation in mice.
Validation from our study: the antibody detects a band at the expected size on western blots, in whole cell lysates and lysosomal-enriched fractions.

CTSB (31718, Cell Signaling)
https://www.cellsignal.com/products/primary-antibodies/cathepsin-b-d1c7y-xp-rabbit-mab/31718
The antibody was validated by the manufacturer for Western blotting, Immunofluorescence, Immunohistochemistry and Flow Cytometry in humans and mice.
Validation from our study: the antibody detects a band at the expected size on western blots, in whole cell lysates and lysosomal-enriched fractions.

CTSD (sc-6486, Santa Cruz)
https://www.scbt.com/p/cathepsin-d-antibody-c-20
The antibody was validated by the manufacturer for Western blotting, Immunofluorescence, Immunohistochemistry and Immunoprecipitation in humans and mice.
Validation from our study: the antibody detects a band at the expected size on western blots, in whole cell lysates and lysosomal-enriched fractions.

CTSK (ab19027, Abcam)
https://www.abcam.com/cathepsin-k-antibody-ab19027.html
The antibody was validated by the manufacturer for Western blotting, Immunofluorescence, Immunohistochemistry in humans and mice.
Validation from our study: the antibody detects a band at the expected size on western blots.

LC3A/B (12741, Cell Signaling)
https://www.cellsignal.com/products/primary-antibodies/lc3a-b-d3u4c-xp-rabbit-mab/12741
The antibody was validated by the manufacturer for Western blotting, Immunofluorescence, Immunohistochemistry and Flow Cytometry in humans and mice.
Validation from our study: the antibody detects a band at the expected size on western blots, in whole cell lysates, and punctate cytoplasmic staining on IF. Additionally, the lipidated, lower migrating form of LC3 was increased in response to treatment with hydroxychloroquine (HCQ) and Chloroquine (CQ), consistent with increased autophagic flux.

GPNMB (38313, Cell Signaling)
https://www.cellsignal.com/products/primary-antibodies/gpnmb-e4d7p-xp-rabbit-mab/38313
The antibody was validated by the manufacturer for Western blotting, Immunohistochemistry and Immunoprecipitation in humans.
Validation from our study: the antibody detects a band at the expected size on western blots.

p62/SQSTM1 (88588, Cell Signaling)
https://www.cellsignal.com/products/primary-antibodies/sqstm1-p62-d5l7g-mouse-mab/88588?site-search-type=Products&N=4294956287&Ntt=sqstm1&fromPage=plp
The antibody was validated by the manufacturer for Western blotting, Immunofluorescence, Immunohistochemistry and Immunoprecipitation in humans.
Validation from our study: the antibody detects a band at the expected size on western blots.

Rab7 (9367, Cell Signaling)
https://www.cellsignal.com/products/primary-antibodies/rab7-d95f2-xp-rabbit-mab/9367
The antibody was validated by the manufacturer for Western blotting, Immunofluorescence, and Immunoprecipitation in humans and mice.
Validation from our study: the antibody detects a band at the expected size on western blots, in whole cell lysates and lysosomal-enriched fractions.

LAMTOR1 (8975, Cell Signaling)
https://www.cellsignal.com/products/primary-antibodies/lamtor1-c11orf59-d11h6-xp-rabbit-mab/8975
The antibody was validated by the manufacturer for Western blotting, Immunofluorescence, Immunohistochemistry and Immunoprecipitation in humans and mice.
Validation from our study: the antibody detects a band at the expected size on western blots, in whole cell lysates and lysosomal-enriched fractions.

LAMTOR2 (8145, Cell Signaling)
https://www.cellsignal.com/products/primary-antibodies/lamtor2-robld3-d7c10-rabbit-mab/8145
The antibody was validated by the manufacturer for Western blotting and Immunoprecipitation in humans and mice.
Validation from our study: the antibody detects a band at the expected size on western blots in whole cell lysates.

LAMTOR3 (8168, Cell Signaling)
https://www.cellsignal.com/products/primary-antibodies/lamtor3-mapksp1-d38g5-rabbit-mab/8168
The antibody was validated by the manufacturer for Western blotting in humans and mice.
Validation from our study: the antibody detects a band at the expected size on western blots in lysosomal-enriched fractions.

FLCN (3697, Cell Signaling)
https://www.cellsignal.com/products/primary-antibodies/flcn-d14g9-rabbit-mab/3697
The antibody was validated by the manufacturer for Western blotting and Immunoprecipitation in humans.
Validation from our study: the antibody detects a band at the expected size on western blots, which was decreased in human cell lines stably transfected with FLCN shRNA. The antibody also detected an expected increase in lysosomal localization on amino acid starvation.

FNIP2 (57612, Cell Signaling)
https://www.cellsignal.com/products/primary-antibodies/fnip2-d3t8z-rabbit-mab/57612
The antibody was validated by the manufacturer for Western blotting and Immunoprecipitation in humans.
Validation from our study: the antibody detects a band at the expected size on western blots. The antibody also detected an

expected increase in lysosomal localization on amino acid starvation.

FNIP2 (HPA042779, Sigma)
https://www.sigmaaldrich.com/US/en/product/sigma/hpa042779
The antibody was validated by the manufacturer for Immunohistochemistry in humans.
Validation from our study: the antibody detects a band at the expected size on western blots which was decreased in human cell lines stably transfected with FNIP2 shRNA.

RagA (4357, Cell Signaling)
https://www.cellsignal.com/products/primary-antibodies/raga-d8b5-rabbit-mab/4357
The antibody was validated by the manufacturer for Western blotting and Immunoprecipitation in humans and mice.
Validation from our study: the antibody detects a band at the expected size on western blots.

RagB (8150, Cell Signaling)
https://www.cellsignal.com/products/primary-antibodies/ragb-d18f3-rabbit-mab/8150
The antibody was validated by the manufacturer for Western blotting and Immunoprecipitation in humans.
Validation from our study: the antibody detects a band at the expected size on western blots.

RagC (5466, Cell Signaling)
https://www.cellsignal.com/products/primary-antibodies/ragc-d31g9-xp-rabbit-mab/5466
The antibody was validated by the manufacturer for Western blotting, Immunohistochemistry and Immunoprecipitation in humans and mice.
Validation from our study: the antibody detects a band at the expected size on western blots.

RagD (4470, Cell Signaling)
https://www.cellsignal.com/products/primary-antibodies/ragd-antibody/4470
The antibody was validated by the manufacturer for Western blotting in humans.
Validation from our study: the antibody detects a band at the expected size on western blots, which was decreased in human cell lines stably transfected with RagD shRNA.

GABARAP (13733, Cell Signaling)
https://www.cellsignal.com/products/primary-antibodies/gabarap-e1j4e-rabbit-mab/13733
The antibody was validated by the manufacturer for Western blotting and Immunofluorescence, in humans and mice.
Validation from our study: the antibody detects a band at the expected size on western blots.

TFEB (A303-673A, Bethyl)
https://www.thermofisher.com/antibody/product/TFEB-Antibody-Polyclonal/A303-673A
The antibody was validated by the manufacturer for Western blotting, Immunohistochemistry and Immunoprecipitation in humans and mice.
Validation from our study: the antibody detects a band at the expected size on western blots, and demonstrated predominantly nuclear localization in human cells (by IF) and mouse renal tumors (by IHC).

TFE3 (PA5-54909, Thermo Fisher Scientific)
https://www.thermofisher.com/antibody/product/TFE3-Antibody-Polyclonal/PA5-54909
The antibody was validated by the manufacturer for Western blotting, Immunofluorescence, Immunohistochemistry in humans and mice.
Validation from our study: the antibody detects a band at the expected size on western blots, and demonstrated predominantly nuclear localization in human cells (by IF) and mouse renal tumors (by IHC). Using this antibody, decreased immunostaining was also observed in human xenograft tissues with genomic deletion of TFE3 via CRISPR-Cas9, by IHC.

TFEB (4240, Cell Signaling)
https://www.cellsignal.com/products/primary-antibodies/tfeb-antibody/4240
The antibody was validated by the manufacturer for Western blotting and Immunoprecipitation in humans.
Validation from our study: the antibody detects a band at the expected size on western blots, and is completely absent in human HEK293T cells with genomic deletion of TFEB via CRISPR-Cas9. Using this antibody, decreased immunostaining was also observed in human xenograft tissues with genomic deletion of TFEB via CRISPR-Cas9, by IHC.

TFE3 (14779, Cell Signaling)
https://www.cellsignal.com/products/primary-antibodies/tfe3-antibody/14779
The antibody was validated by the manufacturer for Western blotting in humans and mice.
Validation from our study: the antibody detects a band at the expected size on western blots, and is significantly decreased in human HEK293T cells with genomic deletion of TFE3 via CRISPR-Cas9.

Phospho-TFEB (S211) (37681, Cell Signaling)
https://www.cellsignal.com/products/primary-antibodies/phospho-tfeb-ser211-e9s8n-rabbit-mab/37681
The antibody was validated by the manufacturer for Western blotting in humans.
Validation from our study: the antibody detects a band at the expected size on western blots, and demonstrated an expected decrease in expression (in response to amino acid starvation, treatment with torin and treatment with lambda phosphatase), and an expected increase in expression (following amino acid stimulation of starved cells), in human HEK293T cells.

Phospho-TFEB (S122) (86843, Cell Signaling)
https://www.cellsignal.com/products/primary-antibodies/phospho-tfeb-ser122-antibody/86843
The antibody was validated by the manufacturer for Western blotting and Immunoprecipitation in humans.
Validation from our study: the antibody detects a band at the expected size on western blots, and demonstrated an expected decreased in expression (in response to amino acid starvation, treatment with torin and treatment with lambda phosphatase), and an expected increase in expression (following amino acid stimulation of starved cells), in human HEK293T cells.

Histone H3 (4499, Cell Signaling)
https://www.cellsignal.com/products/primary-antibodies/histone-h3-d1h2-xp-rabbit-mab/4499
The antibody was validated by the manufacturer for Western blotting, Immunofluorescence, Immunohistochemistry and Flow Cytometry in humans and mice.
Validation from our study: the antibody detects a band at the expected size on western blots, and is present almost exclusively in nuclear fractions of fractionated lysates.

Fibrillarin (2639, Cell Signaling)
https://www.cellsignal.com/products/primary-antibodies/fibrillarin-c13c3-rabbit-mab/2639
The antibody was validated by the manufacturer for Western blotting and Immunofluorescence in humans and mice.
Validation from our study: the antibody detects a band at the expected size on western blots, and is present almost exclusively in nuclear fractions of fractionated lysates.

Lamin A/C (4777, Cell Signaling)
https://www.cellsignal.com/products/primary-antibodies/lamin-a-c-4c11-mouse-mab/4777
The antibody was validated by the manufacturer for Western blotting, Immunofluorescence, Immunohistochemistry and Immunoprecipitation in humans and mice.
Validation from our study: the antibody detects a band at the expected size on western blots, and is present almost exclusively in nuclear fractions of fractionated lysates.

Ki-67 (12202, Cell Signaling)
https://www.cellsignal.com/products/primary-antibodies/ki-67-d3b5-rabbit-mab-mouse-preferred-ihc-formulated/12202
The antibody was validated by the manufacturer for Immunohistochemistry in mice.

GFP (55494, Cell Signaling)
https://www.cellsignal.com/products/primary-antibodies/gfp-5g4-mouse-mab/55494
The antibody was validated by the manufacturer for Immunofluorescence, and Immunoprecipitation in transfected cells.
Validation from our study: the antibody detected exogenous TFEB-GFP and TFE3-GFP in transfected human HEK293T cells, which were predominantly nuclear localized, as expected, by immunofluorescence.

GFP (2956, Cell Signaling)
https://www.cellsignal.com/products/primary-antibodies/gfp-d5-1-rabbit-mab/2956
The antibody was validated by the manufacturer for Western blotting and Immunohistochemistry and Immunoprecipitation in transfected cells.
Validation from our study: the antibody detects a band at the expected size of GFP-tagged proteins on western blots.

HA (3724, Cell Signaling)
https://www.cellsignal.com/products/primary-antibodies/ha-tag-c29f4-rabbit-mab/3724
The antibody was validated by the manufacturer for Western blotting, Immunofluorescence, Immunohistochemistry, Flow Cytometry, ChIP and Immunoprecipitation in in transfected cells.
Validation from our study: the antibody detects a band at the expected size of HA-tagged proteins on western blots.

FLAG (14793, Cell Signaling)
https://www.cellsignal.com/products/primary-antibodies/dykddddk-tag-d6w5b-rabbit-mab-binds-to-same-epitope-as-sigma-s-anti-flag-m2-antibody/14793
The antibody was validated by the manufacturer for Western blotting, Immunofluorescence, Immunohistochemistry, Flow Cytometry, ChIP and Immunoprecipitation in in transfected cells.
Validation from our study: the antibody detects a band at the expected size of FLAG-tagged proteins on western blots.

14-3-3 (pan) (8312, Cell Signaling), 1:1000.
https://www.cellsignal.com/products/primary-antibodies/14-3-3-pan-antibody/8312
The antibody was validated by the manufacturer for Western blotting in humans and mice.
Validation from our study: the antibody detects a band at the expected size on western blots.

Eukaryotic cell lines

Policy information about cell lines

Cell line source(s)

1) All Human embryonic kidney HEK293T cells (parental as well as those with somatic genomic deletion (KO) of TSC1, TSC2 or TSC1/TSC2 via CRISPR-Cas9 genome editing) were a kind gift of TSC Alliance and Dr. Nellist.
2) TRI102 cells derived from a TSC2-null human AML and TRI103 47 cells derived from TRI102 cells stably transfected with wild-type TSC2 (pcDNA3.1 TSC2-zeo) were obtained from ATCC (Manassas, VI) (Catalog numbers: PTA-7368 and PTA-7369).
3) TTJ cells, a Tsc2-null cell line derived from Tsc2+/- C57BL/6 mice, stably transfected with a control vector (TTJ- parental) or wild-type Tsc2 (TTJ-Tsc2) were a kind gift of Dr. Vera Krymskaya

Authentication

All cell lines were authenticated for expresion of TSC1 and TSC2 using immunoblotting and qRT-PCR.

Mycoplasma contamination	Cell lines tested negative for Mycoplasma contamination.
Commonly misidentified lines (See ICLAC register)	N/A

Animals and other organisms

Policy information about studies involving animals; ARRIVE guidelines recommended for reporting animal research

Laboratory animals	Animal Studies: The following strains were used: 1) The Tsc2 +/- A/J mice, heterozygous for a deletion in exons 1-2 were a kind gift from David Kwiatkowski (Harvard University, Boston, USA) 2) Mice carrying loxP sites flanking exon 17 and 18 of Tsc1(Stock Number 005680, Tsc1tm1Djk/J) (The Jackson Laboratory). 3) Mice carrying loxP sites flanking exon 2, 3 and 4 of Tsc2(Stock Number 027458, Tsc2tm1.1Mjgk/J) (The Jackson Laboratory).4) Mice bearing a tamoxifen-inducible Cre recombinase driven by the endogenous mouse Gt(ROSA)26Sor promoter (Stock Number: 004847, R26CreER) (The Jackson Laboratory). 5) Mice heterozygous for the Pax8 cre recombinase knockin gene (Stock Number: 028196, Pax8cre) (The Jackson Laboratory). Conditional deletion of Tsc1 was obtained by tamoxifen treatment of Rosa(ER)-Cre; Tsc1fl/fl mice. Renal tubular-specific deletion of Tsc2 was obtained by crossing heterozygously-expressing Pax8-cre mice with Tsc2fl/wt mice to generate Pax8-Cre; Tsc2 fl/wt mice.<br><br>6-week female NSG (NOD scid gamma) mice for xenograft experiments were obtained from the Johns Hopkins Research Animal Resources (RAR).<br><br>Animal care conditions have been added to the manuscript as follows: Animals will have access to food (standard rodent chow) and water ad libitum. Light will be regulated by timer, with 12 hour on/off cycles. Rooms will be maintained at standard mouse temperature (68-79o F) and humidity (30-70% relative humidity).
Wild animals	No wild animals were used
Field-collected samples	No field-collected samples were used
Ethics oversight	Animal protocols were approved by the JHU Animal Care and Use Committee, under the following protocol:<br>1) The role of the PI3K/mTOR signaling pathways in murine epithelial morphogenesis and migration (MO20M83)<br>2) Targeting Lysosomal Biogenesis in Renal Tumors with TSC1/2 Loss (MO20M185).

Note that full information on the approval of the study protocol must also be provided in the manuscript.
