# Supplementary material for: An mTORC1-mediated negative feedback loop constrains amino acid-induced FLCN-Rag activation in renal cells with TSC2 loss
Source: Nat Commun. 2022 Nov 10;13:6808. doi: 10.1038/s41467-022-34617-7 (PMC9649702; doi:10.1038/s41467-022-34617-7)
Supplement: Supplementary file 4 — Source Data [file 41467_2022_34617_MOESM4_ESM.zip › Source Data blots.pdf]

FIGURE 1 C

CTSK

	HEK WT	TSC1 KO	TSC2 KO	TSC1/2 KO
	1	1.255426	0.979022	1.893069
	1	1.544685	1.463631	2.32944618
	1	2.741359	2.326151	2.908701
	1	1.669231	2.109551	3.30812328
	1	1.041056	1.380428	1.60256958
Mean	1	1.650352	1.651757	2.408381808

RRAGD

	HEK WT	TSC1 KO	TSC2 KO	TSC1/2 KO
	1	1.228785	1.534937	1.870341
	1	1.379655	1.744909	1.712772
	1	1.35928	2.184875	1.737026
	1	1.116357	1.794883	2.026128
	1	0.855676	1.55006	1.206085
Mean	1	1.18795	1.761933	1.71047

UVRAG

	HEK WT	TSC1 KO	TSC2 KO	TSC1/2 KO
	1	1.152277	1.493514	1.820629043
	1	1.692119	2.048185	2.354722417
	1	1.441041	2.068929	2.24487949
	1	1.283718	1.980362	2.82458908
	1	1.043164	1.616281	1.901912665
Mean	1	1.322464	1.841454	2.229346539

FLCN

	HEK WT	TSC1 KO	TSC2 KO	TSC1/2 KO
	1	1.199897	1.081231	1.360391
	1	1.708024	1.446616	1.964078
	1	1.927219	1.698658	1.747458
	1	1.323943	1.863152	2.467916
	1	1.256924	1.529975	1.685991
Mean	1	1.483201	1.523926	1.845167

SQSTM1

	HEK WT	TSC1 KO	TSC2 KO	TSC1/2 KO
	1	1.473464	1.521864	1.790432339
	1	1.940144	1.768861	2.249798821
	1	1.813539	2.407117	2.540985249
Mean	1	1.742382	1.899281	2.193738803

WIPI1

	HEK WT	TSC1 KO	TSC2 KO	TSC1/2 KO
	1	1.942094	3.217478	4.633203
	1	2.701823	3.530009	5.233307
	1	2.111145	3.457442	2.892743
	1	1.143338	2.745289	3.50327
	1	1.198225	3.598932	3.889455
Mean	1	1.819325	3.30983	4.030396

PPARγ

	HEK WT	TSC1 KO	TSC2 KO	TSC1/2 KO
	1	3.27701	3.237807	3.80976761
	1	1.720423	2.903462	1.65257185
	1	1.185551	3.760804	1.70064969
Mean	1	2.060995	3.300691	2.38766305

FIGURE 1G

	Normal	Tumor	Normal	Tumor	Normal	Tumor
	LAMP1		CTSB		CTSD	
	1	3.033684	1	6.372608	1	2.929513
	1	1.11986	1	2.626593	1	5.686796
	1	1.683708	1	1.941206	1	8.4295
	1	1.290636	1	3.370284	1	5.978369
	1	2.693317	1	2.613739		
	1	1.57193				
Mean	1	1.898856	1	3.384886	1	5.756044

	Normal	Tumor	Normal	Tumor	Normal	Tumor
	MCOLN1		ATP6AP2		SQSTM1	
	1	4.883938	1	9.999986	1	9.496876
	1	3.709915	1	6.733236	1	1.074177
	1	3.513741	1	0.731951	1	2.972461
	1	4.30043	1	2.877165	1	1.832529
	1	2.137117	1	2.625252	1	3.667419
	1	1.679675	1	6.339588	1	2.46671
Mean	1	3.370803	1	4.88453	1	3.585029

FIGURE 2A

Nuclear TFEB Fluorescent Intensity	WT	TSC2 KO
	1	1.2
	1	1.5
	1	1.25
	1	1.05
Mean	1	1.25

FIGURE 2B

Nuclear TFE3 Fluorescent Intensity	WT	TSC2 KO
	1	1.59
	1	1.67
	1	1.61
	1	1.81
Mean	1	1.67

FIGURE 2D

Nuclear/ Cytoplasmic TFEB-GFP Fluorescent Intensity	WT	TSC2 KO	TSC2 KO-Rapamycin
	1	1.295583	0.994664
	1	1.401874	1.105434
	1	1.319097	0.975731
	1	1.193592	1.048724
Mean	1	1.3025365	1.03113825

FIGURE 2F

Nuclear/ Cytoplasmic TFEB Band Intensity	HEK WT-dmso	TSC2 KO-dmso	HEK WT rapamycin	TSC2 KO rapamycin
	1	4.663892	0.822477	0.748773
	1.000001	7.647655	0.958056	0.667157
	1	3.981136	0.870872	1.193962
Mean	1.000000333	5.430894333	0.883801667	0.869964

	HEK WT-torin	TSC2 KO torin
	2.222027	3.883065
	2.676757	1.790643
	2.059972	4.432399
Mean	2.319585333	3.368702333

FIGURE 2G

Nuclear/ Cytoplasmic	HEK WT-dmso	TSC2 KO-dmso	HEK WT rapamycin	TSC2 KO rapamycin
TFE3 Band Intensity	1	2.0678	0.3402	0.2484
	1	4.2195	1.3273	0.9856
	0.999962	1.727215	1.191862	1.296567
Mean	0.999987333	2.671505	0.95312067	0.84352233

	HEK WT-torin	TSC2 KO torin
	0.7795	0.4102
	1.7074	1.3497
	1.328917	1.0733
Mean	1.271939	0.9444

FIGURE 2H

Relative Luciferase Units	WT	TSC2 KO
	1	2.9368
	1	1.871909
	1	1.207161
	1	2.089877
Mean	1	2.026437

FIGURE 2J

Nuclear TFE3 H-Score	HEK WT-Xenograft	TSC2 KO-Xenograft
	9.685121224	198.869265
	32.6163373	157.3905834
	32.39499959	136.8637434
	22.9491876	123.0146679
	62.65711386	210.4088781
	60.20361919	169.5544215
		141.9477772
		127.8741186
Mean	36.75106313	158.2404319

FIGURE 2K

Tumor Volume (mm3)	WT - Veh	WT - Torin	WT - Rapa
	1260.98	684.42	303.65
	770.12	893.24	352.14
	509.48	393.03	108.81
	1207.25	117.42	291.24
	1339.03	643.79	81.07
MEAN	1017.37	546.38	227.38

Log [Tumor Volume (mm3)]	WT - Veh	WT - Torin	WT - Rapa
	3.100708198	2.835322692	2.482373285
	2.886558402	2.950968163	2.54671536
	2.70712714	2.594425701	2.03666881
	3.081797214	2.069742076	2.464251022
	3.126790307	2.808744226	1.908860173
MEAN	2.980596252	2.651840572	2.28777373

Tumor Volume (mm3)	T2KO - Veh	T2KO - Torin	T2KO - Rapa
	734.64	660.27	95.37
	1442.51	773.75	80.31
	100.57	1027.53	41.98
	2102.19	985.28	90.31
	1491.14	1039.59	283.53
MEAN	1174.21	897.28	118.30

Log [Tumor Volume (mm3)]	T2KO - Veh	T2KO - Torin	T2KO - Rapa
	2.866074571	2.819721565	1.979411783
	3.159118833	2.888600662	1.904769626
	2.00246845	3.01179451	1.623042434
	3.322671966	2.993559667	1.955735842
	3.17351842	3.016862093	2.452599018
MEAN	2.904770448	2.9461077	1.983111741

FIGURE 3B

	Normal kidney	Tsc2 +/- kidney tumor
	64.88651963	199.374049
	24.88025953	107.991553
	86.19036207	209.3521668
	38.28522077	126.8578573
	35.75434982	142.4186927
	32.08253359	110.3618702
	17.59956444	109.8265565
	21.68133324	129.2394637
	11.70759841	114.0214749
Mean Nuclear TFE3 H-score	37.00752683	138.827076

	Normal kidney	Tsc2 +/- kidney tumor
	57.00335361	168.7488278
	106.9668353	234.6284899
	95.36817944	196.7317375
	50.34453541	162.3567145
	96.07300093	201.4429159
	35.72125073	121.4513762
	26.90465616	137.1861811
	28.60674785	159.4608348
	25.20547812	141.3469127
Mean Nuclear TFEB H-score	58.02155973	169.2615545

FIGURE 4B

Tumor Volume (mm3)	TSC2 KO Control C-1	TFE3 CRISPR 1-1-1	TFE3 CRISPR 3-1-1
	1182.75	4294.51	2800.79
	921.87	3126.59	2482.3
	2195.2	4639.8	2412.52
	2450.5	2644.86	1296.42
	983.22	3857.06	1312.4
Mean	1546.71	3712.56	2060.89

Log [Tumor Volume (mm3)]	TSC2 KO Control C-1	TFE3 CRISPR 1-1-1	TFE3 CRISPR 3-1-1
	3.072892957	3.632913618	3.447280547
	2.964669682	3.495070935	3.394854267
	3.341474094	3.666499261	3.382470922
	3.389254707	3.422402689	3.112745722
	2.992650704	3.586256395	3.118066222
Mean	3.152188429	3.560628579	3.291083536

Tumor Volume (mm3)	TFEB CRISPR B3-1	TFEB CRISPR B4-3-3	DKO T2T33B3-1-11
	1677.59	377.91	496
	3433.64	2177.27	133.41
	4597.18	1740.41	167.28
	4381.2	521.45	148.43
	1375.37	2458.53	358.87
Mean	3093.00	1455.11	260.80

Log [Tumor Volume (mm3)]	TFEB CRISPR B3-1	TFEB CRISPR B4-3-3	DKO T2T33B3-1-11
	3.224685829	2.577388384	2.695481676
	3.53575476	3.337912289	2.125188384
	3.662491509	3.24065157	2.22344402
	3.641593079	2.717212672	2.171521687
	3.138419547	3.390675512	2.554937155
Mean	3.440588945	3.052768085	2.354114585

Tumor Volume (mm3)	DKO T2T31-B3-3
	304.54
	405.06
	328.69
	989.99
	460.58
Mean	497.77

	DKO
Log [Tumor Volume (mm3)]	T2T31-B3-3
	2.483644343
	2.607519358
	2.516786491
	2.995630808
	2.663305075
Mean	2.653377215

FIGURE 5B

p-TFEB (S211)/TFEB	WT	TSC2 KO	TSC2 KO-Rapamycin
	1	0.077933	0.569741
	1	0.395383	1.918291
	1	0.147755	1.398554
	1	0.104977	0.577448
	1	0.056104	1.14113
	1	0.130453	0.386288
Mean	1	0.152100833	0.998575333

p-TFEB (S122)/TFEB	WT	TSC2 KO	TSC2 KO-Rapamycin
	1	0.437627	1.059462
	1	0.251054	1.492941
	1	0.10027	0.853479
	1	0.406264	0.667305
	1	0.351887	0.809382
Mean	1	0.3094204	0.9765138

FIGURE 5D

p-TFEB (S211)/TFEB	WT Xenografts	TSC2 KO Xenografts
	83.06548	15.77375
	98.97809	9.188028
	106.919	7.337736
	95.97537	4.789274
	90.61649	15.2932
		7.856441
Mean	95.110886	10.03973817

FIGURE 6A

Mean Lysosomal Band Intensity	WT	TSC2 KO	TSC2 KO-Rapamycin
RAPTOR	100	31.42959	117.2236
	100	40.51398	97.88595
	100	40.2785	86.17927
Mean	100	37.40735667	100.4296067

Mean Lysosomal Band Intensity	WT	TSC2 KO	TSC2 KO-Rapamycin
TFEB	100	59.33752	79.51032
	100	18.79277	36.85959
	100	53.2048	79.90945
Mean	100	43.77836333	65.42645333

FIGURE 6C

p-TFEB (S211)/TFEB	TSC2 KO-empty	TSC2 KO-RagCGDP (S75L)	TSC2 KO-RagCGTP (Q120L)
	1	2.295437	0.834711
	1	2.194274	0.580057
	1	4.220488	1.595487
Mean	1	2.903399667	1.003418333

p-TFEB (S211)/TFEB	TSC2 KO-empty	TSC2 KO-RagCGDP (S75L)	TSC2 KO-RagCGTP (Q120L)
	1	4.785719	0.680611
	1	4.147446	0.607353
	1	1.902251	0.815353
Mean	1	3.611805333	0.701105667

FIGURE 6D

Nuclear/Cytoplasmic TFEB Band Intensity	HEK293T-empty	TSC2 KO-empty	TSC2 KO-RagCGDP (S75L)	TSC2 KO-RagDGDP (S77L)
	1	2.768628	0.78067	1.112464
	1	2.115387	0.514836	0.404897
Mean	1	2.4420075	0.647753	0.7586805

FIGURE 7C

FLCN/Lamtor1-GFP band intensity	HEK WT-dmso	TSC2 KO-dmso	TSC2 KO rapa	TSC2 KO torin
	1	0.677	0.936	1.211
	1	0.525	0.859	1.097
	1	0.723	1.069	1.086
Mean	1	0.641666667	0.954666667	1.131333333

FNIP2/Lamtor1-GFP band intensity	HEK WT-dmso	TSC2 KO-dmso	TSC2 KO rapa	TSC2 KO torin
	1	0.3665	0.907	1.1377
	1	0.4748	0.7974	0.9014
	1	0.1301	0.5874	0.7676
Mean	1	0.3238	0.763933333	0.935566667

FIGURE 7F

	HEK WT-<br>Wt FLCN	TSC2 KO-<br>Wt FLCN	HEK WT-<br>R164A FLCN	TSC2 KO<br>R164A FLCN
Nuclear/Cytoplasmic TFEB Band Intensity	1.000001	3.739399	3.369457	4.319365
	0.999999	5.636071	2.981436	7.26687
	1	4.348202	3.381379	2.816239
Mean	1	4.574557333	3.244090667	4.800824667

	HEK WT-<br>F118D FLCN	TSC2 KO<br>F118D FLCN	TSC2 KO<br>F118D FLCN+FNIP2
Nuclear/Cytoplasmic TFEB Band Intensity	0.992502	1.280835	0.194756
	1.345155	2.779301	0.933316
	0.440756	0.777597	0.453424
Mean	0.926137667	1.612577667	0.527165333

	HEK WT-<br>Wt FLCN	TSC2 KO-<br>Wt FLCN	HEK WT-<br>R164A FLCN	TSC2 KO<br>R164A FLCN
Nuclear/Cytoplasmic TFE3 Band Intensity	1	3.46727	2.446293	3.280765
	1	1.3999	1.882672	1.587197
	1	3.76539	3.03474	2.888678
Mean	1	2.87752	2.454568333	2.585546667

	HEK WT-<br>F118D FLCN	TSC2 KO<br>F118D FLCN	TSC2 KO<br>F118D FLCN+FNIP2
Nuclear/Cytoplasmic TFE3 Band Intensity	0.572698	0.802257	0.752009
	0.301545	0.651401	0.208398
	0.427654	2.267327	0.397484
Mean	0.433965667	1.240328333	0.452630333

S1A

	GPNMB			
	HEK WT	TSC1 KO	TSC2 KO	TSC1/2 KO
	1	1.533304	11.89551	3.542743
	1	1.9016	8.883881	6.827247
	1	11.01166	43.12875	10.61317
	1	1.668492	12.77078	4.029249
	1	2.971415	18.17096	12.6175
Mean	1	3.817294	18.96998	7.525982

S1D

	WT	TSC2 KO
RFU (328/460 nm)		
	1	1.46
	1	1.26615
	1	1.21
MEAN	1	1.31205

S2D

Nuclear/ Cytoplasmic TFE3-GFP Fluorescent Intensity		
	WT	TSC2 KO
	1	1.074156
	1	1.240326
	1	1.172792
Mean	1	1.162425

S4A

Percentage Confluence	TSC2 KO Control C-1	TFE3 CRISPR 1-1-1	TFE3 CRISPR 3-1-1	TFEB CRISPR B3-1
	92.95993	83.64237	92.74431	92.82065
	76.19018	86.45333	92.10943	92.26823
	82.64404	77.54434	86.62832	90.01475
Mean	83.93	82.55	90.49	91.70

Percentage Confluence	TFEB CRISPR B4-3-3	DKO T33-B3-1-4	DKO T33B3-1-11	DKO T31-B3-3
	34.20604	60.45249	54.89481	36.75994
	34.69344	65.42359	59.77981	34.86794
	30.31629	66.06079	66.61412	28.50622
Mean	33.07	63.98	60.43	33.38

S4B

Absorbance (490 nm)	HEK WT Control-1	TSC2 KO Control C-1	TFE3 CRISPR 1-1-1
	0.956097	1	0.86354
	0.94699	1	0.911404
	0.891791	1	1.090254
Mean	0.93	1.00	0.96

Absorbance (490 nm)	TFE3 CRISPR 3-1-1	TFEB CRISPR B3-1	TFEB CRISPR B4-3-3
	0.763681	0.937621	0.501496
	0.806306	0.924587	0.50945
	0.995103	1.140992	0.871931
Mean	0.86	1.00	0.63

Absorbance (490 nm)	DKO T33-B3-1-4	DKO T33B3-1-11	DKO T31-B3-3
	0.533433	0.604434	0.455569
	0.572877	0.650687	0.433115
	0.736163	1.057199	0.887166
Mean	0.61	0.77	0.59

S6A

RRAGD (FPKM)	**HEK293T**	***TSC* 1,2 KO**
	120.1457	132.0544
	62.54043	169.1923
	118.7739	174.6308
MEAN	100.48668	158.6258333

S6B

RRAGD (FPKM)	**HEK293T**	***TSC*2 KO**	***TSC*2 KO_DKO**
	415.7404	884.5306	437.8484
	569.7385	635.7931	503.7063
	600.1738	738.8208	307.8424
MEAN	528.5509	753.0481667	416.4657

S6C

p-4EBP1(S65)/ T-4EBP1	TSC2 KO-Scr shRNA	TSC2 KO-RRAGD shRNA
	1	0.777126292
	1	0.812444843
	1	0.869229
MEAN	1	0.819600045

p-4EBP1(T37/45)/ T-4EBP1	TSC2 KO-Scr shRNA	TSC2 KO-RRAGD shRNA
	1	0.60690142
	1	0.935674714
	1	0.75831
MEAN	1	0.766962045

p-p70S6K(T389)/ T-p70S6K	TSC2 KO-Scr shRNA	TSC2 KO-RRAGD shRNA
	1	0.636139638
	1	0.524063378
	1	0.865207
MEAN	1	0.675136672

S6D

RRAGD mRNA Fold Change	TSC2 KO Control C-1	TFE3 CRISPR 1-1-1	TFE3 CRISPR 3-1-1	TFEB CRISPR B3-1
	1	0.304866	0.553502	0.439735
	1	0.781494	0.677491	0.839769
	1	0.54976	0.572908	0.817043
MEAN	1	0.545373333	0.601300333	0.698849

RRAGD mRNA Fold Change	TFEB CRISPR B4-3-3	DKO T31-B3-3	DKO T33B3-1-11
	0.203565	0.331496	0.237376
	0.605177	0.486376	0.361577
	0.49066	0.46723	0.309128
MEAN	0.433134	0.428367333	0.302693667

S7E

p-TFEB (S211)-GFP/TFEB-GFP	WT	TSC2 KO
	1	0.602937
	1	0.605975
	1	0.834105
Mean	1	0.681006

p-TFEB (S122)-GFP/TFEB-GFP	WT	TSC2 KO
	1	0.310961
	1	0.715502
	1	0.761192
Mean	1	0.595885

S9E

Tumor Volume (mm3)	Emp	FLCN (R164A)	FLCN (F118D)	FLCN (F118D)+FNIP2
	1994.22	2246.14	1466.19	2334.24134
	1766.55	1530.57	1672.45	984.51045
	3086.87	1644.29	1715	1378.18371
	238.2	3459.75	1115.61	130.038784
	2877.61	1750.02	2763.9	2719.08864
MEAN	1992.69	2126.15	1746.63	1509.21

Log [Tumor Volume (mm3)]	Emp	FLCN (R164A)	FLCN (F118D)	FLCN (F118D)+FNIP2
	3.299773067	3.351436822	3.166190253	3.368145756
	3.247125934	3.184853197	3.223353143	2.99322033
	3.48951834	3.215978416	3.234264124	3.139307112
	2.376941757	3.539044718	3.047512398	2.1140729
	3.459031934	3.243043012	3.441522326	3.434423365
MEAN	3.174478207	3.306871233	3.222568449	3.009833893
